# The impact of self-monitoring in chronic illness on healthcare utilisation: a systematic review of reviews

**DOI:** 10.1186/s12913-015-1221-5

**Published:** 2015-12-18

**Authors:** Hayley McBain, Michael Shipley, Stanton Newman

**Affiliations:** School of Health Sciences, City University London, Northampton Square, London, EC1V 0HB UK; East London NHS Foundation Trust, Trust Headquarters, 9 Alie Street, London, E1 8DE UK; Centre for Rheumatology, University College London Hospitals, 235 Euston Road, Fitzrovia, London, NW1 2BU UK

**Keywords:** Self-monitoring, Chronic illness, Healthcare utilization, Complex interventions, Hospitalization, Systematic review, Meta-analysis

## Abstract

**Background:**

Self-management interventions have been found to reduce healthcare utilisation in people with long-term conditions, but further work is needed to identify which components of these interventions are most effective. Self-monitoring is one such component and is associated with significant clinical benefits. The aim of this systematic review of reviews is to assess the impact of self-monitoring interventions on healthcare utilisation across a range of chronic illnesses.

**Methods:**

An overview of published systematic reviews and meta-analyses. Multiple databases were searched (MEDLINE, CINAHL, PsycINFO, EMBASE, AMED, EBM and HMIC) along with the reference lists of included reviews. A narrative synthesis was performed, accompanied by calculation of the Corrected Cover Area to understand the impact of overlapping primary research papers.

**Results:**

A total of 17 systematic reviews and meta-analyses across three chronic conditions, heart failure, hypertension and chronic obstructive pulmonary disease, were included. Self-monitoring was associated with significant reductions in hospitalisation and re-admissions to hospital.

**Conclusions:**

Self-monitoring has the potential to reduce the pressure placed on secondary care services, but this may lead to increase in services elsewhere in the system. Further work is needed to determine how these findings affect healthcare costs.

**Electronic supplementary material:**

The online version of this article (doi:10.1186/s12913-015-1221-5) contains supplementary material, which is available to authorized users.

## Background

Self-management has been defined as an individual’s ability to manage the clinical and psychosocial consequences, along with the lifestyle changes inherent in living with a chronic condition [1]. Chronic disease self-management interventions, can lead to small, but statistically significant reductions in health service utilisation [2]. These interventions are however, complex in nature as they consist of a number of interacting components [3]. These interacting components, also known as behaviour change techniques [4], are the active ingredients that bring about the desired change in behaviour and improvements in outcomes. In order to optimise the effectiveness of self-management interventions there is a need to determine which specific components of these interventions are most effective in reducing healthcare usage [2].

One such component is self-monitoring, the foundation for self-management [5]. Self-monitoring in the context of chronic illness has been defined as the patient undertaking one or more of the following activities (i) self-measurement of vital signs, symptoms, behaviour or psychological well-being; (ii) self-interpretation of this data; or (iii) self-adjustment of medication, treatment, lifestyle or help-seeking behaviour as a result of self-awareness and/or self-interpretation [6, 7]. The clinical benefits of self-monitoring in chronic illness include reductions in HbA1c [8–12], improvements in blood pressure [13–15], and reductions in mortality and adverse events [16]. As a result, self-monitoring is part of recommended practice in a number of chronic conditions [17–21]. For example National Institute for Health and Care Excellence (NICE) guidelines recommend home blood pressure monitoring in hypertension [17], self-monitoring and self-management of vitamin K antagonists in atrial fibrillation [18] and self-monitoring of blood glucose in diabetes [20, 21]. This has been enabled by more open access to clinical data and the introduction of technology that allows patients to take measurements that would have otherwise required visits to a healthcare setting.

The impact of self-monitoring on healthcare utilisation is however unknown, but nevertheless an important outcome for interventions that are aimed at a population who are likely to require significant healthcare resources [22]. The aim of this systematic review is to assess the impact of interventions whose primary function is self-monitoring, on healthcare utilisation, across a range of chronic conditions.

## Methods

### Inclusion and exclusion criteria

#### Study design

As the volume of literature in this area is large and a number of systematic reviews and meta-analyses have been published, this overview included either systematic reviews or meta-analyses only. Primary research studies were excluded. If the review contained a synthesis of qualitative studies or secondary data (i.e. other systematic reviews or meta-analyses) this content was not extracted. The search was limited to articles in English, but conducted in any country.

#### Population

Adults living with a chronic disease, defined as a physical illness that is prolonged in duration, does not often resolve spontaneously, and is rarely cured completely [23]. Reviews that included more than one long-term physical health condition were excluded in order for data to be summarised within a long-term condition.

#### Intervention

Patient self-monitoring had to be the focus of the review or be an element of all interventions by virtue of the nature of that intervention (i.e. telemonitoring). Self-monitoring was defined as the patient undertaking one or more of the following activities (i) Awareness: Self-measurement of vital signs, symptoms, behaviour or psychological well-being, (ii) Interpretation: Self-interpretation of vital signs, symptoms, behaviour or psychological well-being; or (iii) Response: Self-adjustment of medication, treatment, lifestyle or help-seeking behaviour as a result of self-awareness and/or self-interpretation [6, 7]. Delivered by any method.

#### Outcome

The review had to synthesize the evidence in relation to healthcare utilisation as a primary or secondary outcome. Reviews that were restricted to clinical outcomes, psychosocial outcomes, acceptability, cost-effectiveness or feasibility were excluded from this overview, unless data relating to healthcare utilisation could be extracted.

### Data sources and search strategy

EBSCOHost was used to search CINAHL Plus® full text (1937–2014), MEDLINE with full text (1948 to February 5, 2014) and PsycINFO (from 1806–2014). OVID Online was used to search EMBASE (1996–2014 Week 06), Allied and Complementary Medicine (1985 to February 2014), Evidence Based Medicine Reviews (All) 1 and Health Management Information Consortium (1979 to November 2013). Key words or Medical Subject Headings (MeSH) terms were used, coupled with Boolean logical operators, for both self-monitoring and systematic review.

A full list of search terms, by database, can be found in Additional file 1. Searches were performed in February 2014 and reviews could be have been published at any time. Reference lists of relevant articles were also searched in order to identify additional reviews.

### Review selection

After the removal of duplicates and reviews not published in English, one author assessed all titles for relevance. Those clearly not meeting the inclusion and exclusion criteria were removed and full reviews thought to be of relevance were retrieved for assessment against the inclusion and exclusion criteria. These were assessed by one author and then those judged to be relevant assessed by a second reviewer according to the outlined criteria. Any disagreements were discussed with a third reviewer and resolved by consensus.

### Data extraction

The following characteristics of the reviews were extracted: illness or disease type, self-monitoring activity (i.e. awareness/interpretation/response), type of review, search strategy, inclusion and exclusion criteria, quality assessment, data extraction procedure, total number of studies and participants, author’s conclusions and interpretations. The relevant data were extracted and recorded by one author; independent data extraction was also performed on a random sample of 25 % of reviews by a second reviewer. Any disagreements were then discussed with a third member of the team and resolved by consensus.

### Synthesis

No statistical analysis or meta-analysis were conducted. This review of reviews comprises a narrative synthesis of the available systematic reviews and meta-analyses in this area. For clarity the term ‘primary research studies’ refers to the articles found within the included reviews. As many primary research studies are included in more than one review the overall results and conclusions of an overview can be biased. To assess and understand the potential impact of this overlap, the degree of overlap within and between reviews was measured using the validated Corrected Cover Area (CCA) method [24]. A CCA score of 0–5 is considered slight overlap, 6–10 moderate, 11–15 high and >15 very high [24]. In accordance with reporting guidelines for systematic reviews, a Preferred Reporting Items for Systematic Reviews and Meta-Analyses (PRISMA) [25] checklist can be found in Electronic Additional file 2.

### Review quality

The 11-item Assessment of Multiple Systematic Reviews (AMSTAR) checklist was used to assess the quality of each of the included reviews. The measure possesses satisfactory inter-observer agreement, reliability, construct validity and feasibility [26, 27]. The quality score ranges from 0 (lowest quality) to 11 (highest quality).

## Results

**Fig. 1:**
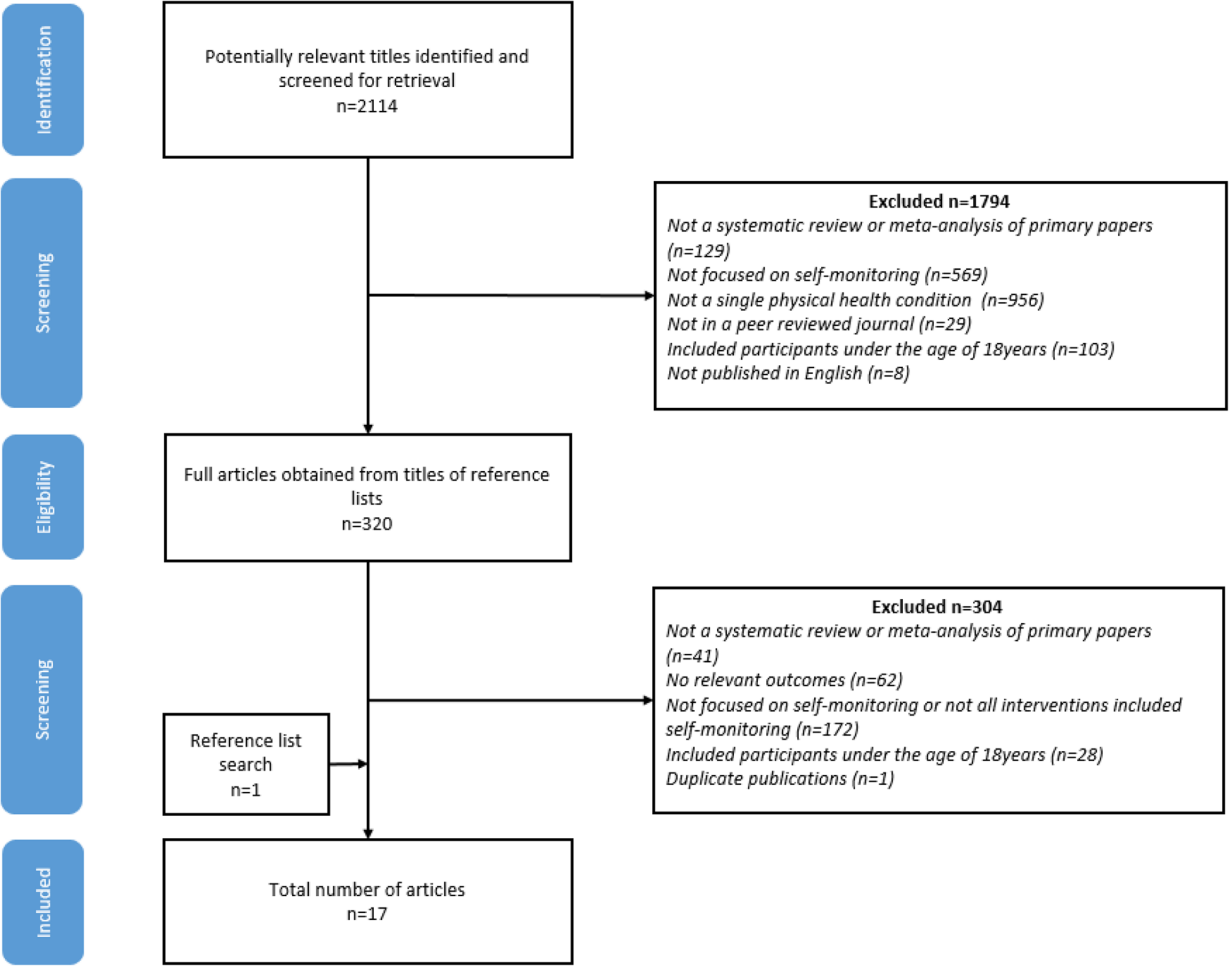
PRISMA Flowchart

A total of 2114 references were retrieved. After exclusions based on title alone 320 full articles were retrieved and after screening 17 articles reporting 16 different systematic reviews or meta-analyses were selected for possible inclusion (Fig. 1). A list of excluded reviews can be found in Additional file 3. One review had been published twice, as a Cochrane review and again as peer reviewed journal article [28, 29]. Both containing the same data, only the journal article was included [28]. One additional review [30] was identified as a result of reference list searches, resulting in a total of 17 reviews in this overview. Of the 320 full text articles reviewed by two authors, there was disagreement on 17 (5.31 %) of these, discussion between the two reviewers resolved 14 of these and 3 were taken to the third reviewer for discussion.

### Review characteristics

The characteristics of the included reviews can be found in a table in Additional file 4. Table 1 provides a summary of this data, with the most frequently evaluated interventions, measures of healthcare utilization and monitored data. The reviews synthesised intervention effectiveness across three chronic conditions; hypertension (*n* = 2), chronic obstructive pulmonary disease (COPD) (*n* = 2) and heart failure (*n* = 13). The overall CCA across all five long-term conditions was 4.10 %, which represented slight overlap [23]. There was however, significant variation between long-term conditions (Table 1). Eight reviews included only randomised controlled trials (RCT), the remaining reviews included a combination of study designs.

**Table 1:** Summary of included systematic reviews and meta-analyses

Condition	No. of reviews	No. of primary research studies	Intervention	Healthcare utilization measure (s)	Monitored data	Purpose of self-monitoring	CCA^a^
Hypertension	2	26	Telemonitoring	GP attendance	Blood pressure	To increase adherence to hypertensive medication, reduce clinical inertia and provide information about the efficacy of treatment in order to alter medication dosage.	15.38 %
COPD	2	15	Action planning & telehealthcare	Hospitalisation, ER visits, GP attendance, discharge to higher levels of care.	Symptoms	PEF is measured and recorded daily in order to adjustment medication.	0 %
Heart failure	13	160	Telemonitoring	Hospitalisation, readmission rates, length of stay, ER visits, home visits, outpatient visits,	Symptoms, weight	Frequent monitoring will allow for early signs and symptoms of decline.	6.67 %

^a^ *CCA* Corrected cover area, ^b^ *INR* International normalized ratio

### Intervention characteristics

Electronic Additional file 5 is a table with detailed information about the characteristics of the interventions. In COPD the interventions were action planning and telehealthcare and in both hypertension and heart failure telemonitoring. Self-monitoring was facilitated through the use of technology in 15 reviews, in which patients took measurements and then transmitted data to a healthcare professional for interpretation and adjustment. Assessing the interventions according to the three components of self-monitoring: awareness, interpretation and response [6, 7]. In only two reviews, one of self-management in heart failure [31] and the other action planning in COPD [32], did interventions consist of all three components. In all other reviews only awareness was achieved.

### Control conditions

On the whole the articles that included studies with a control group generally provided a poor description of the content. Two articles failed to detail the content of any control groups [33, 34]. For a majority there was no consistency in what the intervention group was compared to and for others they were compared to a mixture of usual care and/or an active control group. In most cases however, the definition of usual or standard care was not described or where it was it was not consistent across primary research studies.

### Assessment of review quality

**Fig. 2:**
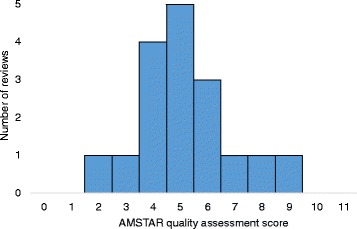
Distribution plot of the quality of review articles

The methodological quality of the 17 reviews varied (Fig. 2), but was generally good (median score = 5). The most common methodological problems were failure to report conflicts of interest, lack of integration of study quality into the conclusions of the review, exclusion of studies based on their publication status, not providing a list of included or excluded studies and not assessing the likelihood of publication bias.

### Intervention effectiveness

Whilst the reviews in heart failure and COPD synthesised the evidence in relation to a range of healthcare utilisation outcomes, the two reviews in hypertension reported the effects in relation to GP attendance only.

#### Hospitalisation

Thirteen of the 17 reviews reported the effects of either telehealth (*n* = 12) or action planning (*n* = 1) on disease-specific and/or all-cause hospitalisation, 11 in heart failure and 2 in COPD.

The one article on action planning was a low quality meta-analysis, in which patients with COPD were actively involved in adjusting their treatment or seeking medical advice in response to their symptoms. This meta-analysis failed to find any significant effect on rates of hospitalisation (*n* = 2, weighted mean difference (WMD) = 0.16, 95 % Confidence Interval (CI) -0.09–0.42) [32] and all of the primary research studies in this review had methodological limitations, increasing the risk of bias.

The reviews of telehealth were more positive. Seven meta-analyses evaluated the evidence with regards to telemonitoring and/or structured telephone support in either COPD or heart failure. The use of technology to support self-monitoring was associated with up to 27 % reduction in total all-cause or disease-specific hospitalisations compared to control conditions (see Table 2 for specific results) [28, 35–40]. All these reviews were rated of moderate or high quality. The quality of the primary research studies within them however, ranged from introducing a low risk of bias [35, 36] to introducing a significant amount of methodological bias [29, 38, 40]. Whilst human-to-human structured telephone support led to a significant reduction in heart failure-related hospitalisations, the one review on human-to-machine structured telephone support failed to have any effect [35]. In contrast to what might be expected, subgroup analyses within this moderate quality review indicated that telemonitoring with medical support available only during office hours was associated with a greater reduction in hospitalisations than when medical support was available 24/7 [35].

**Table 2:** Results of the meta-analyses in relation to hospitalisation for technology enabled self-monitoring

Study	Condition	Comparisons	Results
McLean, 2011	COPD	Telehealthcare versus control	All-cause hospitalisation: OR = 0.46, 95 % CI 0.33–0.65, *p* < 0.00001, *n* = 4
Clark, 2007	Heart failure	TM or STS versus usual care	All-cause hospital admission: STS (RR = 0.94, 95 % CI 0.87 –1.02, *p* = 0.15, *n* = 7). TM (RR = 0.95, 95 % CI 0.89–1.02, *p* = 0.83, *n* = 2). HF-related hospitalization: STS (RR = 0.78, 95 % CI 0.68–0.89, *p* = 0.0003, *n* = 9) TM (RR = 0.79, 95%CI 0.69–0.89, *p* = 0.45, *n* = 1).
Klersy, 2009	Heart failure	RPM versus control	All-cause hospitalisation: RCT (RR = 0.93; 95 % CI 0.73–0.95; *p* = 0.030, *n* = 11) Cohort studies (RR = 0.52; 95 % CI 0.28–0.96; *p* < 0.001, *n* = 3) CHF-related hospitalisations: RCT (RR = 0.71; 95 % CI 0.64–0.80; *p* < 0.001, *n* = 13) when compared with usual care.
Polisena, 2009	Heart failure	TM versus usual care	No of patients hospitalised all-cause: RR = 0.77; 95 % CI 0.65–0.90, *n* = 4
Inglis, 2010	Heart failure	STS or TM versus usual care	All-cause hospitalisation: STS (RR = 0.91, 95 % CI 0.85–0.99, *p* = 0.02, *n* = 11) and TM (RR = 0.92, 95 % CI 0.84–0.99, *p* = 0.02, *n* = 8). CHF-related hospitalisation – STS (RR = 0.77, 95 % CI 0.68–0.87, *p* < 0.0001, *n* = 13) and TM (RR = 0.79, 95 % CI 0.67–0.94, *p* = 0.008, *n* = 4)
Clarke, 2011	Heart failure	TM versus usual care	All-cause hospital admission: RR = 0.99, 95 % CI 0.88–1.11, *p* = 0.84, *n* = 6). CHF-related hospital admission: RR = 0.73, 95 % CI 0.62–0.87, *p* = 0.0004, *n* = 6)
Pandor, 2013	Heart failure	TM with medical support in office hours (TM Office), TM with medical support 24/4 (TM 24/7), Human to machine STS (STS HM) or Human to human STS (STS HH) versus control	All-cause hospitalisation: TM Office (HR: 0.75, 95 % CrI: 0.49–1.10, *p* = NR, *n* = 6). TM 24/7 (HR: 0.81, 95 % CrI: 0.33–2.00, *p* = NR, *n* = 1). STS HM (HR: 1.06, 95 % CrI: 0.44–2.53, *p* = NR, *n* = 1). STS HH (HR: 0.97, 95 % CrI: 0.70, 1.31, *p* = NR, *n* = 9). CHF-related hospitalisation: TM Office (HR: 0.95, 95 % CrI: 0.70, 1.34, *p* = NR, *n* = 3). STS HM (HR: 1.03, 95 % CrI: 0.66, 1.54, *p* = NR, *n* = 1). STS HH (HR: 0.77, 95 % CrI: 0.62, 0.96, *p* = NR, *n* = 8).
Turnock, 2005	COPD	Action planning versus usual care	All-cause hospitalisation: WMD = 0.16, 95 % CI −0.09–0.42, *p* = 0.21, *n* = 2

*TM* telemonitoring, *STS* structured telephone support, *RR* relative risk, *OR* odds ratio, *HR* hazard ratio, *WMD* weighted mean difference, *CI* confidence interval, *Crl* Credible interval, *NR* not reported

Five further systematic reviews, of either low or moderate quality, concluded that there was a positive trend towards a reduction in all-cause and disease-specific hospitalisation in favour of telemonitoring for patients with heart failure [30, 33, 41–43]. Only two of these systematic reviews rated the quality of the primary research studies, both suggested that the studies were of good quality [30, 33].

#### Readmissions

Rates of readmission in heart failure were reported in one meta-analysis and three systematic reviews. The meta-analysis, of moderate quality, found that self-management in which patients were taught to seek medical assistance in response to their symptoms, reduced the odds of all-cause and disease-specific readmission by up to 54 % (*n* = 5, Odds Ratio (OR) = 0.59, 95 % CI 0.44–0.80; *n* = 3, OR = 0.44, 95 % CI 0.27–0.71; respectively) [31]. The quality of the primary research studies included in this review however, varied significantly. Conclusions of the three systematic reviews, which were either of low or moderate quality, indicated an association between telemonitoring and fewer readmissions to hospital [42–44]. As result of the quality of the primary research studies in these systematic reviews the authors concluded that further, more methodological robust trials were needed before widespread adoption of telemonitoring should take place.

#### Length of hospital stay

The conclusions drawn by the authors of eight systematic reviews in heart failure, in relation to the number of days patients spent in hospital, were mixed. Telemonitoring was associated with a reduction in the length of hospital stay in three low to moderate quality systematic reviews; both within the intervention group over time and also when compared to a control group [42–44]. Only one review [44] rated the strength of the evidence, which was considered to be very heterogeneous. The five remaining moderate to high quality systematic reviews failed to find an association between telemonitoring and time spent in hospital [30, 35–37, 40]. All reviews judged the primary research studies to be of least fair quality with a low risk of bias. Except one, which failed to rate quality of the primary research studies [37].

#### Accident and emergency (A & E) attendance

Seven reviews; three meta-analyses and four systematic reviews synthesised the evidence in relation to A & E attendance. Action planning in COPD, which involved patients measuring, interpreting and responding to their data, was not found to have any significant effect on visits to A & E in one meta-analysis (*n* = 2, WMD = −0.01, 95 % CI −0.12–0.10, *p* = 0.85) [32]. However, the primary research studies in this review did include some risk of bias and the review itself was of low quality.

There was a mixed picture in regards to telehealth in heart failure, further muddied by the poor quality of these reviews. Whilst a meta-analysis of telemonitoring in heart failure failed to find any effects on A & E attendance (*n* = 4, Risk Ratio (RR) = 1.04, 95 % CI 0.86–1.26, *p* = 0.67) [37], the systematic reviews in heart failure [30, 40, 42, 43] concluded that telehealth was associated with fewer all-cause and disease-specific A & E visits. Within these reviews the strength of the evidence was either rated as fair [30, 40] or was not rated at all [37, 42, 43]. More promisingly, a moderate quality meta-analysis of telehealth in COPD [28] found that odds of attending the A & E department were significantly reduced in the telehealthcare compared to control group (*n* = 3, OR = 0.27, 95 % CI 0.11–0.66, *p* = 0.005), however, a majority of the primary research studies in the review included significant risk of bias.

#### Outpatient visits

One systematic review, of moderate quality, assessed the impact of telemonitoring in heart failure [40] and concluded that home telemonitoring was associated with increased visits to specialist outpatient services.

#### GP visits

The impact of self-monitoring on the frequency of GP visits was reported in one meta-analysis and two systematic reviews, all rated low quality. The meta-analysis in COPD found no significant difference between action planning and usual care in scheduled (*n* = 1, mean difference (MD) = −0.50, 95 % CI −4.06–3.06, *p* = 0.78) or unscheduled GP visits (*n* = 1, MD = −0.20, 95 % CI −1.55–1.15, *p* = 0.77) [32]. The primary research studies in this review also had a number of methodological limitations. The two systematic reviews in hypertension, one of home-based blood pressure monitoring [34] and the other of telemonitoring [45], both found no impact on GP visits. Neither of these systematic reviews assessed the quality of the primary research studies.

#### Home visits

Two systematic reviews in heart failure reported weak and inconsistent effects for telemonitoring on the frequency of home visits [40, 43]. Although one of these reviews suggested a reduction in home visits, there was no quality assessment of the primary research studies and the review itself was of poor quality [43]. The other systematic review, of moderate quality, concluded that home telemonitoring was associated with a greater number of home care visits [40].

## Discussion

This overview of systematic reviews and meta-analyses has examined the impact of self-monitoring on health service utilisation. It is based on a methodical and extensive literature search, and includes a range of chronic conditions and an assessment of review quality. A total of 17 reviews were synthesized in three long-term conditions: COPD, hypertension and heart failure. In summary, interventions that include self-monitoring can lead to significant reductions in specific areas of healthcare usage, but this is dependent on the chronic illness. An increase in contact with healthcare professionals was also found, specifically in relation to outpatient and home visits. The quality of the primary research studies included in these reviews, and the quality of the reviews themselves was variable and this may potentially bias these conclusions.

Whilst there was evidence to suggest that telemonitoring was associated with fewer visits to A & E, either the reviews themselves were of low quality or the quality of the primary research studies included within them introduced a significant risk of bias, making clear conclusions problematic. The results were more promising in regards to admissions to hospital, which were found to decrease significantly as a result of self-monitoring. These findings were most evident in patients with heart failure, where many primary research studies and reviews had been conducted, but also in COPD. Thus reflecting the findings from the broader self-management literature [2]. In both heart failure and COPD, the significant reductions found in disease-specific and all-cause hospitalisation, and readmission rates, were evidenced only in evaluations of telemonitoring and structured telephone support. Both human-to-human structured telephone support and telemonitoring interventions accompanied by medical support during office hours were found to be particularly advantageous in reducing hospitalization and readmission rates in the short- and long-term. Although the mechanisms of this effect are unclear, technology supported by direct communication with a healthcare professional, rather than automated feedback alone, may allow for more immediate personalised action to be taken, reducing the likelihood of hospitalisation. There was however, significant variation in the quality of the primary research studies included in these reviews. This variation could be attributed in part to the differing methods used to assess study quality, but could also indicate that more methodological robust trials are needed to confirm these findings.

Once admitted to hospital the effects of self-monitoring were only explored in patients with heart failure, and conclusions were inconsistent. Whilst some reviews found that self-monitoring interventions led to a significant reductions in days spent in hospital, others failed to find any such effect. The quality of the reviews themselves, and the primary research studies included within them, suggest that any conclusion that self-monitoring leads to reductions in days spent in hospital are not justified at this time.

Visits to the GP were unaffected by self-monitoring, however the quality of this evidence was poor. It was also unclear if the primary aim of these interventions was to reduce attendance in primary care or to ensure that any reductions in the use of secondary care services did not lead to a redirection of help-seeking in primary care. As visits to primary care were unaffected, it was not possible to test this hypothesis. This overview did however, find that home telemonitoring in heart failure can lead to reductions in hospitalisation and readmission rates, whilst also increasing visits to outpatients and home visits thus suggesting a possible redirection of healthcare usage, rather than an elimination. For any economic assessment, this will only be financially advantageous if the cost of providing care in outpatient services and home visits was less than hospitalisation. Although it is likely that this is the case [46], further exploration of this issue is needed.

In two of the included reviews [31, 32], the interventions focused on patients contacting a healthcare professional in response to their monitored symptoms. This would by definition increase healthcare usage, however the outcomes in both reviews were either hospitalisation or readmission rates suggesting that this additional contact with healthcare services in response to the monitored data was not considered as an outcome, but part of the intervention. Whilst one of these reviews [32] failed to find an effect for action planning on hospital admissions, A & E attendance or GP visits. The significant reduction in disease-specific and all-cause readmission reported in the other review [31] could therefore be a result of a redirection of care to telephone based care, rather than an elimination of healthcare usage. This relationship however, requires further exploration.

The findings of this overview need to be interpreted within the context of the specific chronic condition, as the purpose of self-monitoring, the data monitored and the manner in which healthcare usage was targeted as an outcome, differs between conditions. In hypertension the primary purpose of self-monitoring blood pressure is to increase adherence to hypertensive medication, reduce clinical inertia and provide information about the efficacy of treatment. Guidelines from the European Society of Hypertension [47] suggest that home blood pressure monitoring is suitable for any patient wanting to contribute to their own management. Data is however passed to a healthcare professionals to interpret and if necessary alter medication dosage. As hypertension is managed largely in primary care, these reviews assessed the effects on primary care usage only. In contrast, the guidelines for the management of COPD [48] state that patients at risk of having an exacerbation should be given advice that encourages them to respond promptly to the symptoms of an exacerbation, by starting oral corticosteroids or antibiotic treatment, or adjusting their bronchodilator therapy to control their symptoms. In heart failure, frequent monitoring alerts clinicians to the signs and symptoms of decline, providing the opportunity for intervention prior to the patient becoming seriously ill and needing hospitalisation. Guidelines in the UK and Europe [49–51] recommend that self-monitoring should be part of the treatment of heart failure, by patients monitoring and recognising symptoms, signs and weight gain, and recording daily weight. Patients should then be given the relevant information to know when and how to notify their healthcare professional or self-adjust their diuretic therapy. What is considered vital is an immediate response to these signs and symptoms, which can herald clinical deterioration and thereby avoid hospitalisation. In contrast to the reviews on hypertension which focussed only on primary care as an outcome, the reviews in both COPD and heart failure, primarily focused on the use of secondary care services.

As highlighted elsewhere in the literature [2] it is also important to note that healthcare utilisation is not always patient-led, but often initiated by healthcare professionals. Initiation by a healthcare professional does however, not negate the possibility that self-monitoring can lead to reductions in healthcare usage. Linked to this is the notion that engaging and empowering patients with a chronic condition to adjust their treatment and lifestyle themselves in response to monitored data, may be a more successful route to improving outcomes as opposed to the healthcare professional leading this decision. Although this is a process that has been fostered primarily in COPD treatment guidelines, only one review within this overview [32] explicitly stated that patients with COPD were involved in adjusting their medication and this was only in a subset of the primary research studies. The clinical benefits of empowering patients, rather than healthcare professionals, to interpret their monitored data and adjust accordingly remains uncertain [52–55]. The two reviews in this overview that included interventions with all three components of self-monitoring: awareness, interpretation and adjustment [6, 7] did not exhibit consistent findings. Hence, further work is needed to understand, the potential benefits, in relation to both clinical outcomes and healthcare usage, of patients being involved and leading on adjusting their own treatment in response to monitored data.

It is unlikely that self-monitoring was implemented in isolation within these interventions, however information on the inclusion of other behaviour change techniques were missing. Lack of detail is common in the description of complex interventions, constraining scientific replication and limiting the subsequent introduction of successful interventions [56]. This review has assumed that self-monitoring was an important component of all of the interventions however, without a detailed description of the other behaviour change techniques used, it is not possible to say unequivocally that this was the key behavioural component. Future work would benefit from coding intervention descriptions using the recently developed Behaviour Change Technique Taxonomy (BCTTv1) [4], which enables a detailed description of intervention content according to 93 theoretically derived techniques and may allow for a more systematic analysis of which techniques are more effective in improving outcomes.

Overviews of reviews bring together all of the systematic reviews and meta-analyses in the area in order to provide a summary of the evidence. There are however, several limitations that should be acknowledged. The weight given to heart failure in this overview, as a result of the number of reviews in this area, may limit the generalizability of the results across chronic conditions. As with any overview of secondary data, this review relies on the quality of the reporting found in not only the reviews themselves, but also the primary research studies included within them. The overall quality of the systematic reviews and meta-analyses was good, but with significant variation. In addition a high quality review may contain poor quality evidence, or even limited evidence, because that is all that is available. Therefore, assessment of the quality of the primary research studies within each review was important. Despite this, integration of study quality into the conclusions and recommendations was undertaken in less than half of all reviews and even when quality was assessed in many cases it was evaluated as poor. Finally, the inclusion of primary research studies in more than one systematic review or meta-analyses may have unduly influenced the overall conclusions of this overview. There was however, only slight overlap within this overview, but this did vary quite significantly between chronic conditions, with no overlap in COPD and a very high level of overlap in hypertension. The more diverse the interventions within a condition the lower the degree of overlap. A high degree of overlap may reflect an unnecessary duplication of reviews [24]. Due to the number of retrieved articles one notable limitation to this overview is that only one author reviewed article titles in the first stage of review selection, which may have introduced selection bias.

Despite these limitations, this article attempts to integrate conclusions regarding self-monitoring across a number of long-term conditions and synthesizes both systematic reviews and meta-analyses. The findings of this overview do however, need to be considered in light of the overall quality of the reviews, which varied significantly. Nonetheless it provides a useful synthesis of findings on the role of self-monitoring in chronic illness.

## Conclusions

To accompany the published clinical benefits of self-monitoring, this overview found that self-monitoring can lead to significant reductions in hospitalisation and readmission to hospital, specifically in heart failure and COPD. There was however, evidence to suggest that these reductions may lead to increase in services elsewhere in the healthcare system. Further work is needed to evaluate the extent to which this redirection of services affects healthcare costs. These results need to be interpreted in light of the variation in not only the quality of the systematic reviews themselves, but also the primary research papers included within them, which also varied significantly. Future studies, both primary research studies and reviews, need to better describe intervention content in order to understand the impact of specific components of self-management.

## Additional files

Additional file 1:
**Search strategy.** (PDF 83 kb) Additional file 2:
**PRISMA checklist.** (PDF 89 kb) Additional file 3:
**List of excluded studies.** (PDF 243 kb) Additional file 4:
**Table of review characteristics.** (PDF 132 kb) Additional file 5:
**Table of intervention characteristics.** (PDF 123 kb)

## Abbreviations

A & E: accident and emergency
AMSTAR: Assessment of Multiple Systematic Reviews
BCTTv1: Behaviour Change Technique Taxonomy
CCA: Corrected Cover Area
CI: Confidence Interval
COPD: chronic obstructive pulmonary disease
MD: Mean difference
MeSH: Medical Subject Headings
NICE: National Institute for Health and Care Excellence
OR: Odds Ratio
PRISMA: Preferred Reporting Items for Systematic Reviews and Meta-Analyses
RCT: Randomised controlled trials
RR: Risk Ratio
WMD: Weighted mean difference

## Footnotes

1 ACP Journal Club 1991 to January 2014; Cochrane Register of Controlled Trials January 2014; Cochrane Database of Systematic Reviews 2005 to December 2013, Cochrane Methodology Register 3^rd^ Quarter 2012, Database of Abstracts of Reviews of Effects 1^st^ Quarter 2014, Health Technology Assessment 1^st^ Quarter 2014; NHS Economic Evaluation Database 1^st^ Quarter 2014.
