# Supplementary material for: The impact of self-monitoring in chronic illness on healthcare utilisation: a systematic review of reviews
Source: BMC Health Serv Res. 2015 Dec 18;15:565. doi: 10.1186/s12913-015-1221-5 (PMC4683734; doi:10.1186/s12913-015-1221-5)
Supplement: Additional file 1: — Search strategy. (PDF 83 kb) [file 12913_2015_1221_MOESM1_ESM.pdf]

Electronic Supplementary Material 1

<u>1. Cochrane Library (Title, abstract or keywords)</u>

self care
self monitor*
self administer*
self examin*
self medicat*
self inject*
self evaluat*
self test*
self manage*
self adjust*
self measure*
patient participation
patient monitor*
patient manage*
patient adjust*
patient administer*
patient control*
patient cent?d
telemedicine
telehealth
telecare
telemonitor*
telemetry
home monitor*

<u>2. DARE & HTA</u>

self care
self monitor*
self administer*
self examin*
self medicat*
self inject*
self evaluat*
self test*
self manage*
self adjust*
self measure*
patient participation
Monitoring, Physiologic[Mesh]
patient manage*
patient adjust*
patient administ*
patient control*
patient centered

patient centred
telemedicine
telehealth
telecare
telemonitor*
home monitor*

3. Pub med
self care[Mesh]
self monitor*
self administration[Mesh]
self examination[Mesh]
self medication[Mesh]
self inject*
self test*
self management[Mesh]
self adjust*
self evaluat*
self measure*
patient-centered care[Mesh]
patient participation[Mesh]
Monitoring, Physiologic[Mesh]
patient manage*
patient adjust*
patient administ*
patient control*
telemedicine[Mesh]
telecare
telehealth
telemonitor*
home monitor*

AND

Meta-Analysis[Publication Type][Mesh]
Review[Publication Type][Mesh]
overview
narrative review

5, 6, 7 & 8. AMED, HMIC, EMBASE & PsycINFO
Self Care/
self monitor$.mp.
self administer$.mp.
self examin$.mp.
self medicat$.mp.
self inject$.af
self evaluat$.mp.

self test$.af.
self management.mp.
self adjust$.mp.
self measure$.mp
Patient participation/
Patient monitor$.mp.
Patient manage$.mp.
Patient adjust$.mp.
Patient administer$.mp.
Patient control$.mp.
Patient centred.mp.
Telemedicine/
Telehealth.mp.
Telecare.mp.
telemonitoring.ab. or telemonitoring.ti.
telemetry.mp.
home monitor*

4. CINAHL plus

MH Self Care+
self monitor*
MH Self Administration+
self examine*
MH Self Medication
self inject*
self evaluat*
self test*
self manage*
self adjust*
self measure*
MH Health Services+
MH Monitoring, Physiologic+
patient manage*
patient adjust*
patient administer*
patient control*
MH Telehealth+
telecare
telemonitor*
home monitor*
