# Supplementary material for: The impact of self-monitoring in chronic illness on healthcare utilisation: a systematic review of reviews
Source: BMC Health Serv Res. 2015 Dec 18;15:565. doi: 10.1186/s12913-015-1221-5 (PMC4683734; doi:10.1186/s12913-015-1221-5)
Supplement: Additional file 3: — List of excluded studies. (PDF 243 kb) [file 12913_2015_1221_MOESM3_ESM.pdf]

Electronic Supplementary Material 3

Excluded at stage 2 – included children

Centre for Reviews and Dissemination. The effect of nurse-led diabetes self-management education on glycosylated hemoglobin and cardiovascular risk factors: a meta-analysis (Structured abstract). Database of Abstracts of Reviews of Effects 4. 2013.

Centre for Reviews and Dissemination. The value of self-monitoring of blood glucose: a review of recent evidence (Structured abstract). Database of Abstracts of Reviews of Effects 4. 2013.

Centre for Reviews and Dissemination. The impact of telemedicine interventions involving routine transmission of blood glucose data with clinician feedback on metabolic control in youth with type 1 diabetes: a systematic review and meta-analysis (Provisional abstract). Database of Abstracts of Reviews of Effects 4. 2013.

Centre for Reviews and Dissemination. Electronic media-based health interventions promoting behavior change in youth: a systematic review (Structured abstract). Database of Abstracts of Reviews of Effects 4. 2013.

Centre for Reviews and Dissemination. A systematic review of internet-based self-management interventions for youth with health conditions (Structured abstract). Database of Abstracts of Reviews of Effects 4. 2013.

Centre for Reviews and Dissemination. Health technologies for monitoring and managing diabetes: a systematic review (Structured abstract). Database of Abstracts of Reviews of Effects 4. 2013.

Centre for Reviews and Dissemination. Do school-based asthma education programs improve self-management and health outcomes? (Structured abstract). Database of Abstracts of Reviews of Effects 4. 2013.

Centre for Reviews and Dissemination. The socio-economic impact of telehealth: a systematic review (Provisional abstract). Database of Abstracts of Reviews of Effects 4. 2013.

Centre for Reviews and Dissemination. Beneficial effect of real-time continuous glucose monitoring system on glycemic control in type 1 diabetic patients: systematic review and meta-analysis of randomized trials (Structured abstract). Database of Abstracts of Reviews of Effects 4. 2013.

Centre for Reviews and Dissemination. Efficacy of continuous glucose monitoring in improving glycemic control and reducing hypoglycemia: a systematic review and meta-analysis of randomized trials (Provisional abstract). Database of Abstracts of Reviews of Effects 4. 2013.

Coster S., Gulliford, M.C., Seed, P.T., Royle, P., & Swaminathan, R. 2000. Monitoring blood glucose control in diabetes mellitus:a systematic review. *Health Technology Assessment*, 4, (12)

Cox, N.S., Alison, J.A., Rasekaba, T., & Holland, A.E. Telehealth in cystic fibrosis: A systematic review. *Journal of Telemedicine and Telecare*, 18, (2) March

Farmer, A., Gibson, O.J., Tarassenko, L., & Neil, A. 2005. A systematic review of telemedicine interventions to support blood glucose self-monitoring in diabetes. *Diabetic Medicine*, 22, (10) 1372-1378

George, M. & Topaz, M. 2013. A systematic review of complementary and alternative medicine for asthma self-management. *The Nursing Clinics of North America*, 48, (1) 53-149

Hailey, D., Ohinmaa, A., & Roine, R. 2014. Evidence for the benefits of telecardiology applications: a systematic review (Structured abstract). *Health Technology Assessment Database* (1)
Jaana, M. & Pare, G. 2007. Home telemonitoring of patients with diabetes: A systematic assessment of observed effects. *Journal of Evaluation in Clinical Practice*, 13, (2) 242-253
Jaana, M., Pare, G., & Sicotte, C. 2009. Home telemonitoring for respiratory conditions: A systematic review. *American Journal of Managed Care*, 15, (5) 313-320
Labre, M.P., Herman, E.J., Dumitru, G.G., Valenzuela, K.A., & Cechman, C.L. Public health interventions for asthma: An umbrella review, 1990-2010. *American Journal of Preventive Medicine*, 42, (4) April
Lefevre, F., Piper, M., Weiss, K., Mark, D., Clark, N., & Aronson, N. 2002. Do written action plans improve patient outcomes in asthma? An evidence-based analysis. *The Journal of Family Practice*, 51, (10) 842-848
Marcano Belisario, J.S., Huckvale, K., Greenfield, G., Car, J., & Gunn, L.H. 2013. Smartphone and tablet self management apps for asthma. *Cochrane Database of Systematic Reviews*, 11,
McIntosh, B., Yu, C., Lal, A., Chelak, K., Cameron, C., Singh, S., & Dahl, M. 2010. Efficacy of self-monitoring of blood glucose in patients with type 2 diabetes mellitus managed without insulin: a systematic review and meta-analysis. *Open Medicine*, 4, (2) e102-e113
McLean, S., Chandler, D., Nurmatov, U., Liu, J., Pagliari, C., Car, J., & Sheikh, A. 2011. Telehealthcare for asthma: A Cochrane review. *CMAJ*, 183, (11) 09-E742
Montori, V.M., Helgemoe, P.K., Guyatt, G.H., Dean, D.S., Leung, T.W., Smith, S.A., & Kudva, Y.C. 2004. Telecare for patients with type 1 diabetes and inadequate glycemic control: a randomized controlled trial and meta-analysis. *Diabetes Care*, 27, 1088-1094
Pare, G., Jaana, M., & Sicotte, C. 2007. Systematic review of home telemonitoring for chronic diseases: the evidence base. *Journal of the American Medical Informatics Association*, 14, (3) 269-277 Pare, G., Moqadem, K., Pineau, G., & St-Hilaire, C. 2010. Clinical effects of home telemonitoring in the context of diabetes, asthma, heart failure and hypertension: a systematic review. *Journal of medical Internet research*, 12, (2) e21
St John, A., Davis, W.A., Price, C.P., & Davis, T.M.E. 2010. The value of self-monitoring of blood glucose: a review of recent evidence. *Journal of Diabetes and its Complications*, 24, (2) 129-141
Sutcliffe, P., Martin, S., Sturt, J., Powell, J., Griffiths, F., Adams, A., & Dale, J. 2011. Systematic review of communication technologies to promote access and engagement of young people with diabetes into healthcare. *BMC Endocrine Disorders*, 11,
Yeh, H.-C., Brown, T.T., Maruthur, N., Ranasinghe, P., Berger, Z., Suh, Y.D., Wilson, L.M., Haberl, E.B., Brick, J., Bass, E.B., & Golden, S.H. Comparative effectiveness and safety of methods of insulin delivery and glucose monitoring for diabetes mellitus: A systematic review and meta-analysis. *Annals of Internal Medicine*, 157, (5) 16

Excluded at stage 2 – not a systematic review

Agarwal, R., Bills, J.E., Hecht, T.J.W., & Light, R.P. 2011. Role of home blood pressure monitoring in overcoming therapeutic inertia and improving hypertension control: A systematic review and meta-analysis. *Hypertension*, 57, (1) January-38
Aldcroft, S.A., Taylor, N.F., Blackstock, F.C., & O'Halloran, P.D. 2011. Psychoeducational rehabilitation for ealth behavior change in coronary artery dsease: A systematic review of

controlled trials. *Journal of Cardiopulmonary Rehabilitation and Prevention*, 31, (5) September-October
Alonso-Coello, P., Zhou, Q., & Guyatt, G. Home-monitoring of oral anticoagulation vs. dabigatran: An indirect comparison. *Thrombosis and Haemostasis*, 108, (4) September
Anderson, E. & Esper, G. 2012. A systematic review of teleneurology. *Neurology.Conference: 64th American Academy of Neurology Annual Meeting New Orleans, LA United States.Conference Start: 20120421 Conference End: 20120428.Conference Publication: (var.pagings)*, 78, (1 Meeting Abstract) 22
Ann, M.K., Lokker, C., Handler, S.M., Dolovich, L.R., Holbrook, A.M., O'Reilly, D., Tamblyn, R., Hemens, B.J., Basu, R., Troyan, S., & Roshanov, P.S. 2012. The effectiveness of integrated health information technologies across the phases of medication management: A systematic review of randomized controlled trials. *Journal of the American Medical Informatics Association*, 19, (1) January/February-30
Arad, Y., Fonseca, V., Peters, A., & Vinik, A. 2011. Beyond the monofilament for the insensate diabetic foot: A systematic review of randomized trials to prevent the occurrence of plantar foot ulcers in patients with diabetes. *Diabetes Care*, 34, (4) April-1046
Aspry, K.E., Furman, R., Karalis, D.G., Jacobson, T.A., Zhang, A.M., Liptak, G.S., & Cohen, J.D. Effect of health information technology interventions on lipid management in clinical practice: A systematic review of randomized controlled trials. *Journal of Clinical Lipidology*, 7, (6) November-December
Avery, L., Flynn, D., Van, W.A., Sniehotta, F.F., & Trenell, M.I. Changing physical activity behavior in type 2 diabetes: A systematic review and meta-analysis of behavioral interventions. *Diabetes Care*, 35, (12) December
Aziz, K.M.A. Management of type-1 and type-2 diabetes by insulin injections in diabetology clinics - a scientific research review. *Recent Patents on Endocrine, Metabolic and Immune Drug Discovery*, 6, (2) May
Baradaran, H.R.S. 2010. Effectiveness of diabetes educational interventions in Iran: A systematic review. *Diabetes Technology and Therapeutics*, 12, (4) 317-331
Beatty, L. & Lambert, S. 2013. A systematic review of internet-based self-help therapeutic interventions to improve distress and disease-control among adults with chronic health conditions. *Clinical Psychology Review*, 33, (4) 609-622
Bentsen, S.B., Langeland, E., & Holm, A.L. Evaluation of self-management interventions for chronic obstructive pulmonary disease. *Journal of Nursing Management*, 20, (6) September
Birns, J., Roots, A., & Bhalla, A. Role of telemedicine in the management of acute ischemic stroke. *Clinical Practice*, 10, (2) 2013
Bliziotis, I.A., Destounis, A., & Stergiou, G.S. 2012. Home versus ambulatory and office blood pressure in predicting target organ damage in hypertension: A systematic review and meta-analysis. *Journal of Hypertension*, 30, (7) July-1299
Boland, M.R.S., Tsiachristas, A., Kruis, A.L., Chavannes, N.H., & Rutten-Van Molken, M.P.M.H. The health economic impact of disease management programs for COPD: A systematic literature review and meta-analysis. *BMC Pulmonary Medicine*, 13, (1) 40
Bonner, K., Mezochow, A., Roberts, T., Ford, N., & Cohn, J. Viral load monitoring as a tool to reinforce adherence: A systematic review. *Journal of Acquired Immune Deficiency Syndromes*, 64, (1) 01
Boren, S.A., Wakefield, B.J., Gunlock, T.L., & Wakefield, D.S. 2009. Heart failure self-management education: A systematic review of the evidence. *International Journal of Evidence-Based Healthcare*, 7, (3) 159-168

Bourbeau, J. 2003. Disease-specific self-management programs in patients with advanced chronic obstructive pulmonary disease: a comprehensive and critical evaluation (Structured abstract). *Disease Management and Health Outcomes*, 11, 311-319
Boyde, M., Turner, C., Thompson, D.R., & Stewart, S. 2011. Educational interventions for patients with heart failure: a systematic review of randomized controlled trials. *The Journal of cardiovascular nursing*, 26, (4) 2011-2Aug
Boyers, D., McNamee, P., Clarke, A., Jones, D., Martin, D., Schofield, P., & Smith, B.H. 2013. Cost-effectiveness of self-management methods for the treatment of chronic pain in an aging adult population: a systematic review of the literature. *The Clinical Journal Of Pain*, 29, (4) 366-375
Breland, H.L. & Kamen, D.L. Lupus patient education: An examination of approaches. *International Journal of Clinical Rheumatology*, 7, (5) October
Brewster, L.B. 2010. Systematic Review of the Use of Spot and Overnight Urine for Assessment of Sodium Excretion: 5D.05. *Journal of Hypertension*, Conference, (var.pagings) June
Brouwer, W., Kroeze, W., Crutzen, R., de, N.J., de Vries, N.K., Brug, J., & Oenema, A. 2011. Which intervention characteristics are related to more exposure to internet-delivered healthy lifestyle promotion interventions? A systematic review. *Journal of medical Internet research*, 13, (1) 2011
Bryant, J., McDonald, V.M., Boyes, A., Sanson-Fisher, R., Paul, C., & Melville, J. Improving medication adherence in chronic obstructive pulmonary disease: A systematic review. *Respiratory Research*, 14, (1) 109
Bussey-Smith, K.L. & Rossen, R.D. 2007. A systematic review of randomized control trials evaluating the effectiveness of interactive computerized asthma patient education programs (Structured abstract). *Annals of Allergy, Asthma and Immunology*, 98, 507-516
Carlier, I.V.E., Meuldijk, D., Van Vliet, I.M., Van, F.E., Van Der Wee, N.J.A., & Zitman, F.G. Routine outcome monitoring and feedback on physical or mental health status: Evidence and theory. *Journal of Evaluation in Clinical Practice*, 18, (1) February
Carnes, D., Homer, K.E., Miles, C.L., Pincus, T., Underwood, M., Rahman, A., & Taylor, S.J.C. Effective delivery styles and content for self-management interventions for chronic musculoskeletal pain: A systematic literature review. *Clinical Journal of Pain*, 28, (4) May
Castrejon, I., Silva-Fernandez, L., Bombardier, C., & Carmona, L. 2011. Clinical composite measures of disease activity for diagnosis and followup of undifferentiated peripheral inflammatory arthritis: A systematic review. *Journal of Rheumatology*, 38, (SUPPL.#87) March-53
Catalani, C., Philbrick, W., Fraser, H., Mechael, P., & Israelski, D.M. mHealth for HIV treatment & prevention: A systematic review of the literature. *Open AIDS Journal*, 7, (1) 2013
Centre for Reviews and Dissemination. Communication-related behavior change techniques used in face-to-face lifestyle interventions in primary care: a systematic review of the literature (Structured abstract). Database of Abstracts of Reviews of Effects 4. 2013.
Centre for Reviews and Dissemination. Computer-based education for patients with hypertension: a systematic review (Structured abstract). Database of Abstracts of Reviews of Effects 4. 2013.
Centre for Reviews and Dissemination. Telehealth interventions for the secondary prevention of coronary heart disease: a systematic review (Structured abstract). Database of Abstracts of Reviews of Effects 4. 2013.

Centre for Reviews and Dissemination. Pharmacist interventions to enhance blood pressure control and adherence to antihypertensive therapy: review and meta-analysis (Structured abstract). Database of Abstracts of Reviews of Effects 4. 2013.
Centre for Reviews and Dissemination. A systematic review of randomized trials of disease management programs in heart failure (Structured abstract). Database of Abstracts of Reviews of Effects 4. 2013.
Centre for Reviews and Dissemination. Nurse-led telephone interventions for people with cardiac disease: a review of the research literature (Structured abstract). Database of Abstracts of Reviews of Effects 4. 2013.
Centre for Reviews and Dissemination. A systematic review of psychosocial outcomes following education, self-management and psychological interventions in diabetes mellitus (Structured abstract). Database of Abstracts of Reviews of Effects 4. 2013.
Centre for Reviews and Dissemination. A systematic review of community-based health interventions on depression for older adults with heart disease (Structured abstract). Database of Abstracts of Reviews of Effects 4. 2013.
Centre for Reviews and Dissemination. Systematic review of the chronic care model in chronic obstructive pulmonary disease prevention and management (Structured abstract). Database of Abstracts of Reviews of Effects 4. 2013.
Centre for Reviews and Dissemination. Systematic review: self-management support interventions for irritable bowel syndrome (Structured abstract). Database of Abstracts of Reviews of Effects 4. 2013.
Centre for Reviews and Dissemination. Self-monitoring and other non-pharmacological interventions to improve the management of hypertension in primary care: a systematic review (Structured abstract). Database of Abstracts of Reviews of Effects 4. 2013.
Centre for Reviews and Dissemination. Effectiveness of chronic obstructive pulmonary disease-management programs: systematic review and meta-analysis (Structured abstract). Database of Abstracts of Reviews of Effects 4. 2013.
Centre for Reviews and Dissemination. Effectiveness of chronic care model-oriented interventions to improve quality of diabetes care: a systematic review (Structured abstract). Database of Abstracts of Reviews of Effects 4. 2013.
Centre for Reviews and Dissemination. Educational interventions for migrant South Asians with type 2 diabetes: a systematic review (Structured abstract). Database of Abstracts of Reviews of Effects 4. 2013.
Centre for Reviews and Dissemination. Effectiveness of web-based interventions on patient empowerment: a systematic review and meta-analysis (Provisional abstract). Database of Abstracts of Reviews of Effects 4. 2013.
Centre for Reviews and Dissemination. Self-care and quality of life outcomes in heart failure patients (Structured abstract). Database of Abstracts of Reviews of Effects 4. 2013.
Centre for Reviews and Dissemination. Problem solving in diabetes self-management and control: a systematic review of the literature (Structured abstract). Database of Abstracts of Reviews of Effects 4. 2013.
Centre for Reviews and Dissemination. Evaluation of self-management interventions for chronic obstructive pulmonary disease (Structured abstract). Database of Abstracts of Reviews of Effects 4. 2013.
Centre for Reviews and Dissemination. Psychological intervention following implantation of an implantable defibrillator: a review and future recommendations (Structured abstract). Database of Abstracts of Reviews of Effects 4. 2013.

Centre for Reviews and Dissemination. Heart failure self-management education: a systematic review of the evidence (Structured abstract). Database of Abstracts of Reviews of Effects 4. 2013.
Centre for Reviews and Dissemination. Patient self-administration of medication: a review of the literature (Structured abstract). Database of Abstracts of Reviews of Effects 4. 2013.
Centre for Reviews and Dissemination. Interventions to improve outcomes for minority adults with asthma: a systematic review (Structured abstract). Database of Abstracts of Reviews of Effects 4. 2013.
Centre for Reviews and Dissemination. Interventions to enhance adherence to medications in patients with heart failure: a systematic review (Structured abstract). Database of Abstracts of Reviews of Effects 4. 2013.
Centre for Reviews and Dissemination. Self-management education interventions for persons with schizophrenia: a meta-analysis (Provisional abstract). Database of Abstracts of Reviews of Effects 4. 2013.
Centre for Reviews and Dissemination. Self-management education programs for age-related macular degeneration: a systematic review (Structured abstract). Database of Abstracts of Reviews of Effects 4. 2013.
Centre for Reviews and Dissemination. Self-management education programs for people living with HIV/AIDS: a systematic review (Provisional abstract). Database of Abstracts of Reviews of Effects 4. 2013.
Centre for Reviews and Dissemination. Internet-based physical activity interventions: a systematic review of the literature (Structured abstract). Database of Abstracts of Reviews of Effects 4. 2013.
Centre for Reviews and Dissemination. Self-management interventions for type 2 diabetes: a systematic review (Structured abstract). Database of Abstracts of Reviews of Effects 4. 2013.
Centre for Reviews and Dissemination. Effectiveness of innovations in nurse led chronic disease management for patients with chronic obstructive pulmonary disease: systematic review of evidence (Structured abstract). Database of Abstracts of Reviews of Effects 4. 2013.
Centre for Reviews and Dissemination. Self-management programmes for people post stroke: a systematic review (Provisional abstract). Database of Abstracts of Reviews of Effects 4. 2013.
Centre for Reviews and Dissemination. Effectiveness of diabetes educational interventions in Iran: a systematic review (Structured abstract). Database of Abstracts of Reviews of Effects 4. 2013.
Centre for Reviews and Dissemination. Integrated exercise and self-management programmes in osteoarthritis of the hip and knee: a systematic review of effectiveness (Structured abstract). Database of Abstracts of Reviews of Effects 4. 2013.
Centre for Reviews and Dissemination. Impact of telemedicine intensive care unit coverage on patient outcomes: a systematic review and meta-analysis (Structured abstract). Database of Abstracts of Reviews of Effects 4. 2013.
Centre for Reviews and Dissemination. Nonpharmacological strategies for improving heart failure outcomes in the community: a systematic review (Structured abstract). Database of Abstracts of Reviews of Effects 4. 2013.
Centre for Reviews and Dissemination. A systematic review of variability and reliability of manual and automated blood pressure readings (Structured abstract). Database of Abstracts

of Reviews of Effects 4. 2013.
Centre for Reviews and Dissemination. Relative effectiveness of clinic and home blood pressure monitoring compared with ambulatory blood pressure monitoring in diagnosis of hypertension: systematic review (Structured abstract). Database of Abstracts of Reviews of Effects 4. 2013.
Centre for Reviews and Dissemination. Home versus hospital-based cardiac rehabilitation: a systematic review (Structured abstract). Database of Abstracts of Reviews of Effects 4. 2013.
Centre for Reviews and Dissemination. Home-based pulmonary rehabilitation in chronic obstructive pulmonary disease patients (Structured abstract). Database of Abstracts of Reviews of Effects 4. 2013.
Centre for Reviews and Dissemination. Efficacy of multicomponent treatment in fibromyalgia syndrome: a meta-analysis of randomized controlled clinical trials (Structured abstract). Database of Abstracts of Reviews of Effects 4. 2013.
Centre for Reviews and Dissemination. Can we identify how programmes aimed at promoting self-management in musculoskeletal pain work and who benefits? A systematic review of sub-group analysis within RCTs (Provisional abstract). Database of Abstracts of Reviews of Centre for Reviews and Dissemination. Biomedical informatics applications for asthma care: a systematic review (Structured abstract). Database of Abstracts of Reviews of Effects 4. 2013.
Centre for Reviews and Dissemination. Behavioral treatments of chronic tension-type headache in adults: are they beneficial? (Structured abstract). Database of Abstracts of Reviews of Effects 4. 2013.
Centre for Reviews and Dissemination. Anticoagulation intensity and outcomes among patients prescribed oral anticoagulant therapy: a systematic review and meta-analysis (Structured abstract). Database of Abstracts of Reviews of Effects 4. 2013.
Centre for Reviews and Dissemination. Accuracy of blood-glucose measurements using glucose meters and arterial blood gas analyzers in critically ill adult patients: aystematic review (Provisional abstract). Database of Abstracts of Reviews of Effects 4. 2013.
Chetty, V.T., Almulla, A., Odueyungbo, A., & Thabane, L. 2008. The effect of continuous subcutaneous glucose monitoring (CGMS) versus intermittent whole blood finger-stick glucose monitoring (SBGM) on hemoglobin A1c (HBA1c) levels in type 1 diabetic patients: a systematic review. *Diabetes Research and Clinical Practice*, 81, 79-87
Chodosh, J., Morton, S.C., Mojica, W., Maglione, M., Suttorp, M.J., Hilton, L., Rhodes, S., & Shekelle, P. 2005. Meta-analysis: chronic disease self-management programs for older adults. *Annals of Internal Medicine*, 143, (6) 427
Clark, R.A., Inglis, S.C., McAlister, F.A., Cleland, J.G., & Stewart, S. 2007. Telemonitoring or structured telephone support programmes for patients with chronic heart failure: systematic review and meta-analysis (Structured abstract). *BMJ*, 334, 942
Clarkesmith, D.E., Pattison, H.M., & Lane, D.A. 2013. Educational and behavioural interventions for anticoagulant therapy in patients with atrial fibrillation. *The Cochrane Database Of Systematic Reviews*, 6, CD008600
Cramp, F., Hewlett, S., Almeida, C., Kirwan, J.R., Choy, H.E., Chalder, T., Pollock, J., & Christensen, R. 2013. Non-pharmacological interventions for fatigue in rheumatoid arthritis [Systematic Review]. *Cochrane Database of Systematic Reviews*, 8,
Dantic, D.E. A critical review of the effectiveness of 'teach-back' technique in teaching COPD patients self-management using respiratory inhalers. *Health Education Journal*, 73, (1) January

de, B.J., Legierse, C.M., Prinsen, C.A., & de, K.J. 2011. Patient education in chronic skin diseases: a systematic review. [Review]. *Acta Dermato-Venereologica*, 91, (1) 12-17
DelliFraine, J.L. & Dansky, K.H. 2008. Home-based telehealth : a review and meta-analysis. *Journal of Telemedicine and Telecare* (2) 62-66
Ditewig, J.B., Blok, H., Havers, J., & van Veenendaal, H. 2010. Effectiveness of self-management interventions on mortality, hospital readmissions, chronic heart failure hospitalization rate and quality of life in patients with chronic heart failure: A systematic review. *Patient Education and Counseling*, 78, (3) 297-315
Dolan, G., Smith, L.A., Collins, S., & Plumb, J.M. 2008. Effect of setting, monitoring intensity and patient experience on anticoagulation control: a systematic review and meta-analysis of the literature (Structured abstract). *Current Medical Research and Opinion*, 24, 1459-1472
Dorstyn, D.S., Mathias, J.L., & Denson, L.A. 2011. Psychosocial Outcomes of Telephone-Based Counseling for Adults With an Acquired Physical Disability: A Meta-Analysis. *Rehabilitation Psychology*, 56, (1) February-14
Egginton, J.S., Ridgeway, J.L., Shah, N.D., Balasubramaniam, S., Emmanuel, J.R., Prokop, L.J., Montori, V.M., & Murad, M.H. 2012. Care management for Type 2 diabetes in the United States: a systematic review and meta-analysis. *BMC Health Services Research*, 12, (pp 72) 2012
El-Gayar, O., Timsina, P., Nawar, N., & Eid, W. Mobile applications for diabetes self-management: status and potential. *Journal of diabetes science and technology*, 7, (1) 2013
El-Gayar, O., Timsina, P., Nawar, N., & Eid, W. A systematic review of IT for diabetes self-management: Are we there yet? *International Journal Of Medical Informatics*, 82, (8) August
Ferrier, S., Blanchard, C.M., Vallis, M., & Giacomantonio, N. 2011. Behavioural interventions to increase the physical activity of cardiac patients: A review. *European Journal of Cardiovascular Prevention and Rehabilitation*, 18, (1) February-32
Franek, J. Self-management support interventions for persons with chronic disease: An evidence-based analysis. *Ontario Health Technology Assessment Series*, 13, (9) 2013
Fuchs, S.C., De Mello, R.G.B., & Fuchs, F.C. Home blood pressure monitoring is better predictor of cardiovascular disease and target organ damage than office blood pressure: A systematic review and meta-analysis. *Current Cardiology Reports*, 15, (11) 413
Gaikwad, R.W. 2009. The role of home-based information and communications technology interventions in chronic disease management: A systematic literature review. *Health Informatics Journal*, 15, (2) 122-146
Greaves, C.J., Sheppard, K.E., Abraham, C., Hardeman, W., Roden, M., Evans, P.H., Schwarz, P., & IMAGE Study Group 2011. Systematic review of reviews of intervention components associated with increased effectiveness in dietary and physical activity interventions. *BMC public health*, 11, (pp 119) 2011
Gucciardi, E., Chan, V.W.S., Manuel, L., & Sidani, S. A systematic literature review of diabetes self-management education features to improve diabetes education in women of Black African/Caribbean and Hispanic/Latin American ethnicity. *Patient Education and Counseling*, 92, (2) August
Gupta, V.K., Shobha, P., Maria, A.K., Narang, V.K., Arora, S., Gupta, V., Gupta, M., & Nohria, S. To study the prescription pattern of inhaler devices and medication in management of chronic obstructive pulmonary disease in primary care practice. *Respirology.Conference: Airway Vista 2013 Seoul South Korea.Conference Start: 20130329 Conference End: 20130331.Conference Publication: (var.pagings)*, 18, (pp 22-23) April

Gwadry-Sridhar, F.H., Manias, E., Lal, L., Salas, M., Hughes, D.A., Ratzki-Leewing, A., & Grubisic, M. Impact of interventions on medication adherence and blood pressure control in patients with essential hypertension: A systematic review by the ISPOR medication adherence and persistence special interest group. *Value in Health*, 16, (5) July-August
Haag, G., Diener, H.-C., May, A., Meyer, C., Morck, H., Straube, A., Wessely, P., & Evers, S. 2011. Self-medication of migraine and tension-type headache: Summary of the evidence-based recommendations of the Deutsche Migrane und Kopfschmerzgesellschaft (DMKG), the Deutsche Gesellschaft fur Neurologie (DGN), the Osterreichische Kopfschmerzgesellschaft (OKSG) and the Schweizerische Kopfwehgesellschaft (SKG). *Journal of Headache and Pain*, 12, (2) April-217
Hackam, D.G., Quinn, R.R., Ravani, P., Rabi, D.M., Dasgupta, K., Daskalopoulou, S.S., Khan, N.A., Herman, R.J., Bacon, S.L., Cloutier, L., Dawes, M., Rabkin, S.W., Gilbert, R.E., Ruzicka, M., McKay, D.W., Campbell, T.S., Grover, S., Honos, G., Schiffrin, E.L., Bolli, P., Wilson, T.W., Feldman, R.D., Lindsay, P., Hill, M.D., Gelfer, M., Burns, K.D., Vallee, M., Prasad, G.V.R., Lebel, M., McLean, D., Arnold, J.M.O., Moe, G.W., Howlett, J.G., Boulanger, J.-M., Larochelle, P., Leiter, L.A., Jones, C., Ogilvie, R.I., Woo, V., Kaczorowski, J., Trudeau, L., Petrella, R.J., Milot, A., Stone, J.A., Drouin, D., Lavoie, K.L., Lamarre-Cliche, M., Godwin, M., Tremblay, G., Hamet, P., Fodor, G., Carruthers, S.G., Pylypchuk, G.B., Burgess, E., Lewanczuk, R., Dresser, G.K., Penner, S.B., Hegele, R.A., McFarlane, P.A., Sharma, M., Reid, D.J., Tobe, S.W., Poirier, L., & Padwal, R.S. The 2013 Canadian hypertension education program recommendations for blood pressure measurement, diagnosis, assessment of risk, prevention, and treatment of hypertension. *Canadian Journal of Cardiology*, 29, (5) May
Han, H.-R., Song, H., Nguyen, T., & Kim, M. Measuring self-care in hypertensive patients: A systematic review of literature. *Journal of Clinical Hypertension.Conference: American Society of Hypertension, Inc*, 27th, (var.pagings) April
Hesselbjerg, L.J., Pedersen, H.S., Asmussen, M.B., & Petersen, K.D. Is dabigatran considered a cost-effective alternative to warfarin treatment: A review of current economic evaluations worldwide. *Journal of Medical Economics*, 16, (7) July
Hoeks, L., Greven, W., & de Valk, H. Real-time continuous glucose monitoring system for treatment of diabetes: a systematic review (Structured abstract). Diabetic Medicine 28[4], 386-394. 2011.
Huckvale, K., Car, M., Morrison, C., & Car, J. 2012. Apps for asthma self-management: A systematic assessment of content and tools. *BMC Medicine*, 10,
Huibers, L., Smits, M., Renaud, V., Giesen, P., & Wensing, M. 2011. Safety of telephone triage in out-of-hours care: A systematic review. [References]. *Scandinavian Journal of Primary Health Care* (4) 198-209
Iversen, M.D. 2012. Rehabilitation interventions for pain and disability in osteoarthritis: A review of interventions including exercise, manual techniques, and assistive devices. *American Journal of Nursing*, 112, (3 SUPPL.#1) March-S37
Jang, Y. & Yoo, H. Self-management programs based on the social cognitive theory for Koreans with chronic diseases: A systematic review. *Contemporary Nurse*, 40, (2) February
Janssen, V., De, G., V, Dusseldorp, E., & Maes, S. Lifestyle modification programmes for patients with coronary heart disease: A systematic review and meta-analysis of randomized controlled trials. *European Journal of Preventive Cardiology*, 20, (4) August
Jaramillo, A., Welch, V.A., Ueffing, E., Gruen, R.L., Bragge, P., Lyddiatt, A., & Tugwell, P. 2013. Prevention and self-management interventions are top priorities for osteoarthritis systematic reviews. *Journal Of Clinical Epidemiology*, 66, (5) 503-510 available from:

http://search.ebscohost.com/login.aspx?direct=true&db=mnh&AN=22995854&site=ehost-live
Jia, C.E., Zhang, H.P., Lv, Y., Liang, R., Jiang, Y.Q., Powell, H., Fu, J.J., Wang, L., Gibson, P.G., & Wang, G. The asthma control test and asthma control questionnaire for assessing asthma control: Systematic review and meta-analysis. *Journal of Allergy and Clinical Immunology*, 131, (3) March
Johansson, T. & Wild, C. 2014. Telemedicine in Stroke Management. Systematic Review (Structured abstract). *Health Technology Assessment Database* (1)
Jonsdottir, H. Self-management programmes for people living with chronic obstructive pulmonary disease: A call for a reconceptualisation. *Journal of Clinical Nursing*, 22, (5-6) March
Kruis, A.L., Smidt, N., Assendelft, J.W., Gussekloo, J., Boland, R.M., Ruttenvan Molken, M., & Chavannes, N.H. 2013. Integrated disease management interventions for patients with chronic obstructive pulmonary disease [Systematic Review]. *Cochrane Database of Systematic Reviews*, 10,
Kuethe, M.C., VaessenVerberne, A.A., Elbers, R.G., & Van Aalderen, M.W. 2013. Nurse versus physician-led care for the management of asthma [Systematic Review]. *Cochrane Database of Systematic Reviews*, 2,
Lami, M.J., Martinez, M.P., & Sanchez, A.I. Systematic review of psychological treatment in fibromyalgia. *Current Pain and Headache Reports*, 17, (7) 345
Lawrence, M., Booth, J., Mercer, S., & Crawford, E. A systematic review of the benefits of mindfulness-based interventions following transient ischemic attack and stroke. *International Journal of Stroke*, 8, (6) August
Lee, T.W., Lee, S.H., Kim, H.H., & Kang, S.J. Effective intervention strategies to improve health outcomes for cardiovascular disease patients with low health literacy skills: A systematic review. *Asian Nursing Research*, 6, (4) December
Lehman, A., Yohannes, S., & MacDonald, C. 2011. Moving from patient-centred to family-centred care? A systematic review of psycho-educational programs for people and partners affected by arthritis. *Journal of Rheumatology.Conference: 2nd Mexican-Canadian Congress of Rheumatology Cancun Mexico.Conference Start: 20110210 Conference End: 20110215.Conference Publication: (var.pagings)*, 38, (6) June
Lemmens, K.M.M., Lemmens, L.C., Boom, J.H.C., Drewes, H.W., Meeuwissen, J.A.C., Steuten, L.M.G., Vrijhoef, H.J.M., & Baan, C.A. 2011. Chronic care management for patients with COPD: A critical review of available evidence. *Journal of Evaluation in Clinical Practice*, 19, (5) October
Linn, A.J., Vervloet, M., van, D.L., Smit, E.G., & Van Weert, J.C. 2011. Effects of eHealth interventions on medication adherence: a systematic review of the literature. [Review]. *Journal of medical Internet research*, 13, (4) e103
Liu, H., Li, S., & Feldman, M.W. 2012. Forced bachelors, migration and HIV transmission risk in the context of China's gender imbalance: A meta-analysis. *AIDS Care*, 24, (12) 1487-1495
Loveman, E., Frampton, G., & Clegg, A.J. 2008. The clinical effectiveness of diabetes education models for type 2 diabetes: a systematic review. *Health Technology Assessment*, 12, (9) 1-136
Lu, Z., Cao, S., Chai, Y., Liang, Y., Bachmann, M., Suhrcke, M., & Song, F. 2012. Effectiveness of interventions for hypertension care in the community - a meta-analysis of controlled studies in China. *BMC Health Services Research*, 12, 216

Ludwig, W., Wolf, K.-H., Duwenkamp, C., Gusew, N., Hellrung, N., Marschollek, M., Wagner, M., & Haux, R. Health-enabling technologies for the elderly - An overview of services based on a literature review. *Computer Methods and Programs in Biomedicine*, 106, (2) May
Mahtani, K.R., Heneghan, C.J., Nunan, D., Bankhead, C., Keeling, D., Ward, A.M., Harrison, S.E., Roberts, N.W., Hobbs, R.F., & Perera, R. 2012. Optimal loading dose of warfarin for the initiation of oral anticoagulation [Systematic Review]. *Cochrane Database of Systematic Reviews*, 12,
Mazze, R., Akkerman, B., & Mettner, J. 2011. An overview of continuous glucose monitoring and the ambulatory glucose profile. *Minnesota medicine*, 94, (8) Aug-44
McDermott, M.n.S. & While, A.E. 2013. Maximizing the healthcare environment: a systematic review exploring the potential of computer technology to promote self-management of chronic illness in healthcare settings. *Patient Education and Counseling*, 92, (1) 13-22
McLean, S., Chandler, D., Nurmatov, U., Liu, J., Pagliari, C., Car, J., & Sheikh, A. 2011. Telehealthcare for asthma: A Cochrane review. *CMAJ*, 183, (11) 09-E742
McLean, S., Chandler, D., Nurmatov, U., Liu, J., Pagliari, C., Car, J., & Sheikh, A. 2010. Telehealthcare for asthma. *McLean.Susannah., Chandler.David., Nurmatov.Ulugbek., Liu Joseph., Pagliari.Claudia., Car.Josip., Sheikh.Aziz.Telehealthcare.for asthma.Cochrane Database of Systematic Reviews: Reviews 2010 Issue 10 John.Wiley.& Sons., Ltd.Chichester, UK DOI.: 10.1002./*
Merolli, M., Gray, K., & Martin-Sanchez, F. Health outcomes and related effects of using social media in chronic disease management: A literature review and analysis of affordances. *Journal of Biomedical Informatics*, 46, (6) December
Millard, T., Elliott, J., & Girdler, S. Self-management education programs for people living with HIV/AIDS: A Systematic Review. *AIDS Patient Care and STDs*, 27, (2) 01
Minet, L.M. 2010. Mediating the effect of self-care management intervention in type 2 diabetes: A meta-analysis of 47 randomised controlled trials. *Patient Education and Counseling*, 80, (1) 29-41
Molloy, G.J., O'Carroll, R.E., Witham, M.D., & McMurdo, M.E.T. 2012. Interventions to enhance adherence to medications in patients with heart failure a systematic review. *Circulation: Heart Failure*, 5, (1) January-133
Monninkhof E.Van Der Valk 2003. Self-management education for patients with chronic obstructive pulmonary disease: A systematic review. *Thorax*, 58, (5) 394-398
Mosa, A.S., Yoo, I., & Sheets, L. A systematic review of healthcare applications for smartphones. *BMC medical informatics and decision making*, 12, (pp 67) 2012
Muller-Barna, P., Schwamm, L.H., & Haberl, R.L. 2012. Telestroke increases use of acute stroke therapy. *Current Opinion in Neurology*, 25, (1) February-10
Nakao, M.Y. 2003. Blood pressure-lowering effects of biofeedback treatment in hypertension: A meta-analysis of randomized controlled trials. *Hypertension Research*, 26, (1) 37-46
Neubeck, L.R. 2009. Telehealth interventions for the secondary prevention of coronary heart disease: A systematic review. *European Journal of Cardiovascular Prevention and Rehabilitation*, 16, (3) 281-289
Niiranen, T.J., Thijs, L., Asayama, K., Johansson, J.K., Ohkubo, T., Kikuya, M., Boggia, J., Hozawa, A., Sandoya, E., Stergiou, G.S., Tsuji, I., Jula, A.M., Imai, Y., & Staessen, J.A. 2012. The International Database of HOme blood pressure in relation to Cardiovascular Outcome

(IDHOCO): moving from baseline characteristics to research perspectives. *Hypertension Research: Official Journal Of The Japanese Society Of Hypertension*, 35, (11) 1072-1079
Oliveira, C., Simoes, M., Carvalho, J., & Ribeiro, J. Combined exercise for people with type 2 diabetes mellitus: A systematic review. *Diabetes Research and Clinical Practice*, 98, (2) November
Oliveira, V.C., Ferreira, P.H., Maher, C.G., Pinto, R.Z., Refshauge, K.M., & Ferreira, M.L. Effectiveness of self-management of low back pain: Systematic review with meta-analysis. *Arthritis Care and Research*, 64, (11) November
Olsson, L.E. Efficacy of person-centred care as an intervention in controlled trials a systematic review.
Omboni, S., Gazzola, T., Carabelli, G., & Parati, G. Clinical usefulness and cost effectiveness of home blood pressure telemonitoring:Meta-analysis of randomized controlled studies. *Journal of Hypertension*, 31, (3) 2013
Oosterom-Calo, R., Van Ballegooijen, A.J., Terwee, C.B., Te Velde, S.J., Brouwer, I.A., Jaarsma, T., & Brug, J. Determinants of heart failure self-care: A systematic literature review. *Heart Failure Reviews*, 17, (3) May
Peeters, J.M., Wiegers, T.A., & Friele, R.D. How technology in care at home affects patient self-care and self-management: A scoping review. *International Journal of Environmental Research and Public Health*, 10, (11) 29
Polisena, J., Tran, K., Cimon, K., Hutton, B., McGill, S., & Palmer, K. 2009. Home telehealth for diabetes management: A systematic review and meta-analysis. *Diabetes, Obesity and Metabolism*, 11, (10) 913-930
Polisena, J.T. 2010. Home telehealth for chronic obstructive pulmonary disease: A systematic review and meta-analysis. *Journal of Telemedicine and Telecare*, 16, (3) 120-127
Press, V.G., Pappalardo, A.A., Conwell, W.D., Pincavage, A.T., Prochaska, M.H., & Arora, V.M. 2012. Interventions to improve outcomes for minority adults with asthma: A systematic review. *Journal of General Internal Medicine*, 27, (8) August-1015
Primack, B.A., Carroll, M.V., McNamara, M., Klem, M.L., King, B., Rich, M., Chan, C.W., & Nayak, S. Role of video games in improving health-related outcomes: A systematic review. *American Journal of Preventive Medicine*, 42, (6) June
Purc-Stephenson, R.J. & Thrasher, C. Patient compliance with telephone triage recommendations: A meta-analytic review. *Patient Education and Counseling*, 87, (2) May
Radhakrishnan, K. The efficacy of tailored interventions for self-management outcomes of type 2 diabetes, hypertension or heart disease: A systematic review. *Journal of Advanced Nursing*, 68, (3) March
Rao, G., Burke, L.E., Spring, B.J., Ewing, L.J., Turk, M., Lichtenstein, A.H., Cornier, M.-A., Spence, J.D., & Coons, M. 2011. New and emerging weight management strategies for busy ambulatory settings: A scientific statement from the american heart association: Endorsed by the society of behavioral medicine. *Circulation*, 124, (10) 06-1203
Ridner, S.H., Fu, M.R., Wanchai, A., Stewart, B.R., Armer, J.M., & Cormier, J.N. 2012. Self-management of lymphedema: A systematic review of the literature from 2004 to 2011. *Nursing Research*, 61, (4) July-August
Rosse, B.A., Vowle, K.E., Keogh, E., Eccleston, C., & Mountain, G.A. 2009. Technologically-assisted behaviour change: A systematic review of studies of novel technologies for the management of chronic illness. *Journal of Telemedicine and Telecare*, 15, (7) 327-338
Rubin, M.N., Wellik, K.E., Channer, D.D., & Demaerschalk, B.M. 2013. A systematic review of telestroke. *Postgraduate Medicine*, 125, (1) 45-50 available from:

http://search.ebscohost.com/login.aspx?direct=true&db=mnh&AN=23391670&site=ehost-live
Russell-Minda E.Jutai 2009. Health technologies for monitoring and managing diabetes: a systematic review. *Journal of diabetes science and technology*, 3, (6) 1460-1471
Schmidt-Hansen, M., Baldwin, D.R., & Hasler, E. 2012. What is the most effective follow-up model for lung cancer patients? A systematic review. *Journal of Thoracic Oncology*, 7, (5) 2012-2824
Smith, J.R., Mugford, M., Holland, R., Noble, M.J., & Harrison, B.D.W. 2007. Psycho-educational interventions for adults with severe or difficult asthma: A systematic review. *Journal of Asthma*, 44, (3) 219-241
Solomon, M.R. 2008. Information technology to support self-management in chronic care: A systematic review. *Disease Management & Health Outcomes*, 16, (6) 391-401 Steinsbekk, A., Rygg, L., Lisulo, M., Rise, M.B., & Fretheim, A. 2012. Group based diabetes self-management education compared to routine treatment for people with type 2 diabetes mellitus. A systematic review with meta-analysis. *BMC Health Services Research*, 12, 213
Stellefson, M., Dipnarine, K., & Stopka, C. The chronic care model and diabetes management in US primary care settings: a systematic review. *Preventing chronic disease*, 10, (pp E26) Feb
Stellefson, M., Chaney, B., Barry, A.E., Chavarria, E., Tennant, B., Walsh-Childers, K., Sriram, P.S., & Zagora, J. 2013. Web 2.0 chronic disease self-management for older adults: a systematic review. *Journal of medical Internet research*, 15, (2) e35 Stoilkova, A., Janssen, D.J.A., & Wouters, E.F.M. Educational programmes in COPD management interventions: A systematic review. *Respiratory Medicine*, 107, (11) November
Sutcliffe, P., Martin, S., Sturt, J., Powell, J., Griffiths, F., Adams, A., & Dale, J. 2011. Systematic review of communication technologies to promote access and engagement of young people with diabetes into healthcare. *BMC Endocrine Disorders*, 11,
Thongsai, S. & Youjaiyen, M. 2013. The long-term impact of education on diabetes for older people: a systematic review. *Global Journal Of Health Science*, 5, (6) 30-39
Thorpe, C.T., Fahey, L.E., Johnson, H., Deshpande, M., Thorpe, J.M., & Fisher, E.B. Facilitating Healthy Coping in Patients With Diabetes: A Systematic Review. *Diabetes Educator*, 39, (1) January-February
Vieira, D.S.R. 2010. Home-based pulmonary rehabilitation in chronic obstructive pulmonary disease patients. *Current Opinion in Pulmonary Medicine*, 16, (2) 134-143
Viswanathan, M., Golin, C.E., Jones, C.D., Ashok, M., Blalock, S.J., Wines, R.C.M., Coker-Schwimmer, E.J.L., Rosen, D.L., Sista, P., & Lohr, K.N. Interventions to improve adherence to self-administered medications for chronic diseases in the United States: A systematic review. *Annals of Internal Medicine*, 157, (11) 2012
Wagner, F., Basran, J., & Dal Bello-Haas, V. 2012. A review of monitoring technology for use with older adults. *Journal of Geriatric Physical Therapy*, 35, (1) January-March
Ward, A.M., Takahashi, O., Stevens, R., & Heneghan, C. 2012. Home measurement of blood pressure and cardiovascular disease: systematic review and meta-analysis of prospective studies. [Review]. *Journal of Hypertension*, 30, (3) 449-456
Wilcox, M.E. & Adhikari, N.K.J. The effect of telemedicine in critically ill patients: systematic review and meta-analysis. *Critical Care*, 16, (4) R127
Williams, J.L.S., Walker, R.J., Smalls, B.L., Campbell, J.A., & Egede, L.E. Effective interventions to improve medication adherence in Type 2 diabetes: A systematic review. *Diabetes Management*, 4, (1) January

Wilson, C., Alam, R., Latif, S., Knighting, K., Williamson, S., & Beaver, K. Patient access to healthcare services and optimisation of self-management for ethnic minority populations living with diabetes: A systematic review. *Health and Social Care in the Community*, 20, (1) January
Wofford, J.L.W. 2008. Best strategies for patient education about anticoagulation with warfarin: a systematic review. *BMC Health Services Research*, 8, (pp 40) 2008
Yehle, K.S. & Plake, K.S. 2010. Self-efficacy and educational interventions in heart failure: A review of the literature. *Journal of Cardiovascular Nursing*, 25, (3) 175-188

<u>Excluded at stage 2 – no relevant outcomes</u>

Allemann, S., Houriet, C., Diem, P., & Stettler, C. 2009. Self-monitoring of blood glucose in non-insulin treated patients with type 2 diabetes: A systematic review and meta-analysis. *Current Medical Research and Opinion*, 25, (12) 2903-2913
Andalusian Agency for Health Technology Assessment 2014. Telemedicine application in Andalusia - systematic review (Structured abstract). *Health Technology Assessment Database* (1)
Andalusian Agency for Health Technology Assessment 2014. Telemedicine. Applications in emergencies - systematic review (Structured abstract). *Health Technology Assessment Database* (1)
Baron, J., McBain, H., & Newman, S. 2012. The impact of mobile monitoring technologies on glycosylated hemoglobin in diabetes: a systematic review. *Journal of diabetes science and technology*, 6, (5) 1185-1196
Bartoli, L.Z. 2009. Systematic review of telemedicine services for patients affected by chronic obstructive pulmonary disease (COPD). *Telemedicine and e-Health*, 15, (9) 877-883
Belanger, E., Bartlett, G., Dawes, M., Rodriguez, C., & Hasson-Gidoni, I. Examining the evidence of the impact of health information technology in primary care: An argument for participatory research with health professionals and patients. *International Journal Of Medical Informatics*, 81, (10) October
Bensink, M., Hailey, D., & Wootton, R. 2006. A systematic review of successes and failures in home telehealth: Preliminary results. *Journal of Telemedicine and Telecare*, 12, (Suppl 3) 8-16
Bensink, M., Hailey, D., & Wootton, R. 2007. A systematic review of successes and failures in home telehealth. Part 2: Final quality rating results. *Journal of Telemedicine and Telecare*, 13, (Suppl 3) 10-14
Bloomfield HE, Krause A, Greer N et al. Meta-analysis: effect of patient self-testing and self-management of long-term anticoagulation on major clinical outcomes. *Ann Intern Med* 2011;154:472-482
Bray, E.P.H., Holder, R., Mant, J., & McManus, R.J. 2010. Does self-monitoring reduce blood pressure? Meta-analysis with meta-regression of randomized controlled trials. *Annals of Medicine*, 42, (5) 371-386
Cappuccio, F.P., Kerry, S.M., Forbes, L., & Donald, A. 2004. Blood pressure control by home monitoring - Meta-analysis of randomised trials. *British Medical Journal*, 329, (7464) 499
Carral, S.F., Sanchez, P., & Lizan, L. Costs analysis of a mobile phone telemonitoring system for glycaemic control in patients with diabetes mellitus (DM) in spain: Preliminary results. *Value in Health.Conference: ISPOR 15th Annual European Congress Berlin*

Germany.Conference Start: 20121103 Conference End: 20121107.Conference Publication: (var.pagings), 15, (7) November
Centre for Reviews and Dissemination. The clinical and cost-effectiveness of patient education models for diabetes: a systematic review and economic evaluation (Structured abstract). Database of Abstracts of Reviews of Effects 4. 2013.
Centre for Reviews and Dissemination. Promoting the use of personal asthma action plans: a systematic review (Structured abstract). Database of Abstracts of Reviews of Effects 4. 2013.
Centre for Reviews and Dissemination. Home blood pressure measurement: a systematic review (Structured abstract). Database of Abstracts of Reviews of Effects 4. 2013.
Centre for Reviews and Dissemination. Using the common sense model of self-regulation to review the effects of self-monitoring of blood glucose on glycemic control for non-insulin-treated adults with type 2 diabetes (Provisional abstract). Database of Abstracts of Reviews of Effects 4. 2013.
Centre for Reviews and Dissemination. Telecare for patients with type 1 diabetes and inadequate glycemic control: a randomized controlled trial and meta-analysis (Provisional abstract). Database of Abstracts of Reviews of Effects 4. 2013.
Centre for Reviews and Dissemination. Home telemonitoring for type 2 diabetes: an evidence-based analysis (Provisional abstract). Database of Abstracts of Reviews of Effects 4. 2013.
Centre for Reviews and Dissemination. Comparative analysis of the efficacy of continuous glucose monitoring and self-monitoring of blood glucose in type 1 diabetes mellitus (Provisional abstract). Database of Abstracts of Reviews of Effects 4. 2013.
Centre for Reviews and Dissemination. Effects of self-management health information technology on glycaemic control for patients with diabetes: a meta-analysis of randomized controlled trials (Provisional abstract). Database of Abstracts of Reviews of Effects 4. 2013.
Centre for Reviews and Dissemination. Effect of mobile phone intervention for diabetes on glycaemic control: a meta-analysis (Structured abstract). Database of Abstracts of Reviews of Effects 4. 2013.
Centre for Reviews and Dissemination. Mediating the effect of self-care management intervention in type 2 diabetes: a meta-analysis of 47 randomised controlled trials (Structured abstract). Database of Abstracts of Reviews of Effects 4. 2013.
Centre for Reviews and Dissemination. Glycaemic control in type 1 diabetes during real time continuous glucose monitoring compared with self monitoring of blood glucose: meta-analysis of randomised controlled trials using individual patient data (Structured abstract). Database of Abstracts of Reviews of Effects 4. 2013.
Centre for Reviews and Dissemination. Effect of study setting on anticoagulation control: a systematic review and metaregression (Structured abstract). Database of Abstracts of Reviews of Effects 4. 2013.
Centre for Reviews and Dissemination. Changes in home versus clinic blood pressure with antihypertensive treatments: a meta-analysis (Structured abstract). Database of Abstracts of Reviews of Effects 4. 2013.
Chambers S.Chadda 2010. How much does international normalized ratio monitoring cost during oral anticoagulation with a vitamin K antagonist? A systematic review. *International Journal of Laboratory Hematology*, 32, (4) 427-442
Christensen, T.D. & Larsen, T.B. Precision and accuracy of point-of-care testing coagulometers used for self-testing and self-management of oral anticoagulation therapy. *Journal of Thrombosis and Haemostasis*, 10, (2) February

Christensen, T.D., Johnsen, S.P., Hjortdal, V.E., & Hasenkam, J.M. 2007. Self-management of oral anticoagulant therapy: A systematic review and meta-analysis. *International Journal of Cardiology*, 118, (1) 54-61
Ciere, Yvette, Martin Cartwright, and Stanton P. Newman. "A systematic review of the mediating role of knowledge, self-efficacy and self-care behaviour in telehealth patients with heart failure." *Journal of telemedicine and telecare* 18.7 (2012): 384-391.
Connock M, Stevens C, Fry-Smith A et al. Clinical effectiveness and cost-effectiveness of different models of managing long-term oral anticoagulation therapy: a systematic review and economic modelling. *HTA* 2007;11.
Clar C, Barnard K, Cummins E, Royle P, Waugh N. Self-monitoring of blood glucose in type 2 diabetes: Systematic review. *HTA* 2010;14.
Coster S., Gulliford, M.C., Seed, P.T., Powrie, J.K., & Swaminathan, R. 2000. Self-monitoring in type 2 diabetes mellitus: A meta-analysis. *Diabetic Medicine*, 17, (11) 755-761
Faas, A., Schellevis, F.G., & Van-Eijk, J.T. 1997. The efficacy of self-monitoring of blood glucose in NIDDM subjects: a criteria-based literature review. *Diabetes Care*, 20, 1482-1486
Farmer, A.J., Perera, R., Ward, A., Heneghan, C., Oke, J., Barnett, A.H., Davidson, M.B., Garcia-Alamino JM, Ward AM, Alonso-Coelle P, Bankhead C, Fitzmaurice D, Heneghan C. Self-monitoring and self-management of oral anticoagulation. *Cochrane Database Syst Rev* 2010.
Guerci, B., Coates, V., Schwedes, U., & O'Malley, S. Meta-analysis of individual patient data in randomised trials of self monitoring of blood glucose in people with non-insulin treated type 2 diabetes. *BMJ (Online)*, 344, (7847) e486
Heneghan, C., Alonso-Coello, P., Garcia-Alamino, J.M., Perera, R., Meats, E., & Glasziou, P. 2006. Self-monitoring of oral anticoagulation: a systematic review and meta-analysis. *Lancet*, 367, (9508) 404-411
Heneghan, C.P. 2007. Assessing differential attrition in clinical trials: self-monitoring of oral anticoagulation and type II diabetes. *BMC Medical Research Methodology*, 7, (pp 18) 2007
Holmes, V. & Griffiths, P. 2002. Self-monitoring of glucose levels for people with type 2 diabetes. *British journal of community nursing*, 7, (1) 41-46
Jansen, J.P. 2006. Self-monitoring of glucose in type 2 diabetes mellitus: A Bayesian meta-analysis of direct and indirect comparisons. *Current Medical Research and Opinion*, 22, (4) 671-681
Kleefstra N, Hortensius J, van Hateren KJJ et al. Self-monitoring of blood glucose in noninsulin-treated type 2 diabetes: an overview. *Diabetes Metab Syndr Obes* 2009;2:155-163.
Malanda UL, Welschen LM, Riphagen II, Dekker JM, Nijpels G, Bot SD. Self-monitoring of blood glucose in patients with type 2 diabetes mellitus who are not using insulin. *Cochrane Database Syst Rev* 2012.
McAndrew, L., Schneider, S.H., Burns, E., & Leventhal, H. 2007. Does patient blood glucose monitoring improve diabetes control?: A systematic review of the literature. *Diabetes Educator*, 33, (6) 991-1011
McGeoch G, Derry S, Moore RA. Self-monitoring of blood glucose in type-2 diabetes: what is the evidence? *Diabetes Metab Res Rev* 2007;23:423-440.
Mistry, H. Systematic review of studies of the cost-effectiveness of telemedicine and telecare. Changes in the economic evidence over twenty years. *Journal of Telemedicine and Telecare*, 18, (1) January

Ogedegbe, G. & Schoenthaler, A. 2006. A systematic review of the effects of home blood pressure monitoring on medication adherence. *Journal of Clinical Hypertension*, 8, (3) 174-180
Pare, G., Poba-Nzaou, P., & Sicotte, C. Home telemonitoring for chronic disease management: An economic assessment. *International Journal of Technology Assessment in Health Care*, 29, (2) April
Polisena, J.C. 2009. Home telehealth for chronic disease management: A systematic review and an analysis of economic evaluations. *International Journal of Technology Assessment in Health Care*, 25, (3) 339-349
Poolsup, N., Suksomboon, N., & Jiamsathit, W. 2008. Systematic Review of the Benefits of Self-Monitoring of Blood Glucose on Glycemic Control in Type 2 Diabetes Patients. *Diabetes technology & therapeutics*, 10, (s1) S51-S66
Poolsup, N., Suksomboon, N., & Rattanasookchit, S. 2009. Meta-analysis of the benefits of self-monitoring of blood glucose on glycemic control in type 2 diabetes patients: an update. *Diabetes technology & therapeutics*, 11, (12) 775-784
Powell, H. & Gibson, P.G. 2002. Options for self-management education for adults with asthma. *Cochrane Database of Systematic Reviews* (3)
Renda, S.M. 2006. A practical look at self-monitoring of blood glucose. *Insulin*, 1, (4) 141-147
Sarol Jr, J.R., Nicodemus Jr, N.A., Tan, K.M., & Grava, M.B. 2005. Self-monitoring of blood glucose as part of a multi-component therapy among non-insulin requiring type 2 diabetes patients: A meta-analysis (1966-2004). *Current Medical Research and Opinion*, 21, (2) 173-183
Saudek, C.D., Derr, R.L., & Kalyani, R.R. 2006. Assessing glycemia in diabetes using self-monitoring blood glucose and hemoglobin A1c. *JAMA : the journal of the American Medical Association*, 295, (14) 1688-1697
Siebenhofer A, Berghold A, Sawicki PT. Systematic review of studies of self-management of oral anticoagulation. *Thromb Haemost* 2004;91:225-232.
Song, M. & Lipman, T.H. 2008. Concept analysis: Self-monitoring in type 2 diabetes mellitus. *International journal of nursing studies*, 45, (11) 1700-1710
Towfigh, A., Romanova, M., Weinreb, J.E., Munjas, B., Suttorp, M.J., Zhou, A., & Shekelle, P.G. 2008. Self-monitoring of blood glucose levels in patients with type 2 diabetes mellitus not taking insulin: A meta-analysis. *American Journal of Managed Care*, 14, (7) 468-475
Uhlig, K., Patel, K., Ip, S., Kitsios, G.D., & Balk, E.M. 2013. Self-measured blood pressure monitoring in the management of hypertension: a systematic review and meta-analysis. *Annals Of Internal Medicine*, 159, (3) 185-194
Verberk, W.J., Kessels, A.G.H., & Thien, T. 2011. Telecare is a valuable tool for hypertension management, a systematic review and meta-analysis. *Blood Pressure Monitoring*, 16, (3) June-155
Verberk, W.J., Kessels, A.G.H., & Thien, T. 2011. Telecare is a valuable tool for hypertension management, a systematic review and meta-analysis. *Blood Pressure Monitoring*, 16, (3) June-155
Verberk, W.J.K. 2005. Home blood pressure measurement: A systematic review. *Journal of the American College of Cardiology* , 46, (5) 743-751
Ward, A.M., Takahashi, O., Stevens, R., & Heneghan, C. 2012. Home measurement of blood pressure and cardiovascular disease: systematic review and meta-analysis of prospective studies. [Review]. *Journal of Hypertension*, 30, (3) 449-456

Welschen LM, Bloemendal E, Nijpels G et al. Self-monitoring of blood glucose in patients with type 2 diabetes who are not using insulin: a systematic review. *Diabetes Care* 2005;28:1510-1517.
Welschen LM, Bloemendal E, Nijpels G et al. Self-monitoring of blood glucose in patients with type 2 diabetes mellitus who are not using insulin. *Cochrane Database Syst Rev* 2005.
Willems, D.C.M. 2006. Cost-effectiveness of self-management in asthma: A systematic review of peak flow monitoring interventions. *International Journal of Technology Assessment in Health Care*, 22, (4) 436-442
Xu, Z., Wang, Z., Ou, J., Xu, Y., Yang, S., & Zhang, X. Two monitoring methods of oral anticoagulant therapy in patients with mechanical heart valve prothesis: A meta-analysis. *Journal of Thrombosis and Thrombolysis*, 33, (1) January
