# Supplementary material for: The impact of self-monitoring in chronic illness on healthcare utilisation: a systematic review of reviews
Source: BMC Health Serv Res. 2015 Dec 18;15:565. doi: 10.1186/s12913-015-1221-5 (PMC4683734; doi:10.1186/s12913-015-1221-5)
Supplement: Additional file 4: — Table of review characteristics. (PDF 132 kb) [file 12913_2015_1221_MOESM4_ESM.pdf]

Electronic Supplementary Material 4

Table Review characteristics

First author year	Type of review	Aim	Period searched	Inclusion/Exclusion criteria	n primary research studies(n participants)	Primary outcome/s	Quality assessment tool	AMSTAR score
Hypertension								
Jaana 2007	SR	Evaluate the evidence for the effectiveness of hypertension telemonitoring.	1966 to 2006	Design: Experimental or quasi-experimental<br>Participants: Chronic hypertension<br>Intervention: Home telemonitoring	14(1119)	BP	None	4
AbuDagga 2010	SR & MA	What is the impact of BP telemonitoring technologies on clinical, patient-centred outcomes, healthcare utilization & cost?	1995 to September 2009	Design: RCT, single group, quasi-experimental<br>Participants: Established diagnosis of uncontrolled hypertension<br>Intervention: Telemonitoring	15(3192)	BP	None	4
COPD								

First author year	Type of review	Aim	Period searched	Inclusion/Exclusion criteria	n primary research studies(n participants)	Primary outcome/s	Quality assessment tool	AMSTAR score
Turnock 2005	SR & MA	Determine whether action plans alone, or as part of a broader self-management intervention, are an effective way to manage COPD exacerbations.	Not stated	Design: RCT<br>Participants: Diagnosed primary COPD<br>Intervention: Action planning	3(367)	Hospital admissions	Jadad scale & Cochrane	4
McLean 2012	SR & MA	To review the effectiveness of telehealthcare for COPD compared with face-to-face usual care in improving quality of life & reducing accident & emergency department visits & hospitalisations.	Inception to January 2010	Design: RCT<br>Participants: Diagnosed COPD<br>Intervention: Telehealthcare	10(1307)	Quality of life, healthcare utilisation	Cochrane risk of bias	5
Heart failure								

First author year	Type of review	Aim	Period searched	Inclusion/Exclusion criteria	n primary research studies(n participants)	Primary outcome/s	Quality assessment tool	AMSTAR score
Louis 2003	SR	To present the current evidence on telemonitoring as a means of reducing hospitalisations in heart failure.	1996 to 2002	Design: NR<br>Participants: Diagnosed HF<br>Intervention: Telemonitoring	24(3643)	Acceptability, hospital admissions	None	4
Jovicic 2006	SR & MA	Determine the effectiveness of self-management interventions on hospital readmission rates, mortality & QoL in patients diagnosed with heart failure.	1966 to Nov 2005	Design: RCT<br>Participants: Hospitalised with diagnosed HF<br>Intervention: Self-management	6(857)	Readmissions, mortality	Author designed	7
Martinez 2006	SR	To assess the value of home monitoring for heart failure patients.	1966 to Apr-04	Design: Experimental studies<br>Participants: Not stated<br>Intervention: Home monitoring	42(2303)	Patient acceptability	Jovell & Navarro-Rubio scale	6

First author year	Type of review	Aim	Period searched	Inclusion/Exclusion criteria	n primary research studies(n participants)	Primary outcome/s	Quality assessment tool	AMSTAR score
Chaudhry 2007	SR	In-depth examination of a wide range of telemonitoring interventions in heart failure to find out which components are effective.	1966 to Aug-06	Design: Randomized study design<br>Participants: Adults with HF<br>Intervention: Home monitoring	9(3582)	Hospitalization, mortality	Juni et al & York Centre for Reviews & Dissemination	5
Clark 2007	SR & MA	To determine whether remote monitoring without regular clinic or home visits improves outcomes for patients with CHF.	Jan 2002 to May 2006	Design: RCT<br>Participants: HF, living at home<br>Intervention: Remote monitoring	14(4264)	Hospitalization, mortality	Cochrane	8
Dang 2009	SR	Examine the evidence base for home telehealth & remote monitoring interventions in the management of heart failure.	1966 to Apr-09	Design: RCT<br>Participants: HF<br>Intervention: Home telehealth & remote monitoring	9(2017)	Hospitalization, mortality	Adapted Hailey et al which was adapted from Jovell & Navarro-Rubio scale	5

First author year	Type of review	Aim	Period searched	Inclusion/Exclusion criteria	n primary research studies(n participants)	Primary outcome/s	Quality assessment tool	AMSTAR score
Klersy 2009	SR & MA	Assess the effect of remote patient monitoring on the outcome of CHF patients.	Jan 2000 to October 2008	Design: RCT, observational cohort studies<br>Participants: CHF, living at home<br>Intervention: Remote patient monitoring	32(8612)	Hospitalization, mortality	CONSORT & STROBE	6
Maric 2009	SR	Review studies conducted in heart failure telemonitoring, which are not telephone based, but utilize the concept of patient self-monitoring in heart failure.	Inception to August 2007	Design: Any<br>Participants: HF<br>Intervention: Use of technology or device to assist in self-monitoring	56(NR)	Hospitalization	None	2

First author year	Type of review	Aim	Period searched	Inclusion/Exclusion criteria	n primary research studies(n participants)	Primary outcome/s	Quality assessment tool	AMSTAR score
Polisena 2010	SR & MA	Conduct a SR & MA to look at clinical outcomes, patient QoL & the use of healthcare services for home telemonitoring compared to usual care for patients with CHF.	1998 to 2008	Design: RCT, observational studies<br>Participants: CHF<br>Intervention: Home telemonitoring	22(3028)	Hospitalization, mortality	Adapted version Jovell & Navarro-Rubio scale	5
Inglis 2010	SR & MA	To review RCTs of structured telephone support or telemonitoring compared to standard practice for patients with CHF.	From 2006 onwards	Design: RCT<br>Participants: CHF, within the community<br>Intervention: Structured telephone support or telemonitoring	30(10490)	Hospitalization, mortality	Author designed	9
Clarke 2011	MA	To assess the effectiveness of telemonitoring on primary & secondary outcomes.	From January 1969 to October 2009	Design: RCT<br>Participants: CHF<br>Intervention: Telemonitoring	13(NR)	Hospitalization, mortality	None	5

**First author year**	**Type of review**	**Aim**	**Period searched**	**Inclusion/Exclusion criteria**	**n primary research studies(n participants)**	**Primary outcome/s**	**Quality assessment tool**	**AMSTAR score**
Giamouzis 2012	SR	To assess whether telemonitoring provides any substantial benefit in patients with heart failure.	Inception to November 2011	Design: RCT<br>Participants: CHF<br>Intervention: Telemonitoring	12(3877)	Mortality, healthcare utilisation	None	3
Pandor 2013	SR & MA	To determine whether RM strategies improve outcomes for adults who have been recently discharged (<28 days) following an unplanned admission due to heart failure.	2008 to January 2012 (plus papers from 2 earlier reviews)	Design: RCT, observational cohort studies<br>Participants: Adults, HF, discharged from hospital with <28days<br>Intervention: Remote monitoring	21(6317)	All-cause mortality	Criteria based on The Delphi list & The Newcastle-Ottawa Scale	6

AMSTAR – Assessing the Methodological Quality of Systematic Reviews; BP – Blood Pressure; CHF - Chronic Heart Failure; CONSORT - Consolidated Standards of Reporting Trials; COPD - Chronic Obstructive Pulmonary Disease; HF- Heart Failure; MA – Meta-Analysis; NR – Not Reported; QoL – Quality of Life; RCT – Randomised Controlled Trial; SR – Systematic Review; STROBE - Strengthening the Reporting of Observational studies in Epidemiology; RM – remote monitoring
