# Supplementary material for: The impact of self-monitoring in chronic illness on healthcare utilisation: a systematic review of reviews
Source: BMC Health Serv Res. 2015 Dec 18;15:565. doi: 10.1186/s12913-015-1221-5 (PMC4683734; doi:10.1186/s12913-015-1221-5)
Supplement: Additional file 5: — Table of intervention characteristics. (PDF 123 kb) [file 12913_2015_1221_MOESM5_ESM.pdf]

Electronic Supplementary Material 5

Table. Intervention characteristics

**First author, year**	**Type of intervention**	**Level of patient involvement**	**Recommended frequency of monitoring**	**Behaviour/s monitored**	**Follow-up time**
Hypertension					
Jaana, 2007	BP telemonitoring	Data was given to a HCP. No information on what these readings were used for.	Range 6 a day to weekly	BP, heart rate, symptoms, medication adherence, stress, ECG, weight, sleep quality	Study duration range 5 days-12 months
AbuDagga, 2010	Telemonitoring	Self-measurement & self-transmission of BP. Typically, when BP values exceeded predetermined parameters, an alarm message was automatically generated & sent to a nurse or pharmacist, who contacted patients with disease management tips &/or contacted primary care providers for action. In other cases, the alarm message was sent directly to patients with instructions to contact their physicians.	Range once a week to a few times a day	BP (in some studies titration decisions, side effects)	Study duration range 8 weeks-24 months
COPD					

First author, year	Type of intervention	Level of patient involvement	Recommended frequency of monitoring	Behaviour/s monitored	Follow-up time
Turnock, 2005	Action planning	All studies included patients using an action plan to interpret symptoms & make adjustments to their medications or seeking HCP advice.	NR	Symptoms	6 months & 1 year
McLean, 2012	Telehealthcare	In all studies patients were required to transmit data to HCP for personalised feedback. In some studies patients were trained to make changes to their medication according to an action plan.	NR	NR	3, 6 & 12 months
Heart failure					
Louis, 2003	Telemonitoring	NR	NR	Weight, BP, HR, ECG, respiratory rate, body temperature, extracellular fluid, weight, O2 saturation	Range post intervention-12 months
Jovicic, 2006	Self-management	Patients were taught to recognise when to seek medical assistance.	NR	Sign, symptoms, weight	Range 3 months-1 year
Martinez-Everss, 2006	Home monitoring	Data was given to a HCP. There is no information on what these readings were used for.	NR	ECG, weight, BP, symptoms	Range 3-12 months
Chaudhry, 2007	Telemonitoring	Data was given to a HCP & was used by a nurse or clinician to adjust medication.	Range twice daily to being determined by patient status	Symptoms, weight, physiologic measures	Range 60 days-12 months

First author, year	Type of intervention	Level of patient involvement	Recommended frequency of monitoring	Behaviour/s monitored	Follow-up time
Clark, 2007	Telemonitoring or structured telephone support	Data was given to a HCP. There is no information on what these readings were used for.	Range daily to being determined by patient status	Symptoms, weight, pulse, BP, electrocardiographic data	Range 2 months-400 days
Dang, 2009	Home telehealth remote monitoring	Data was given to a HCP. There is no information on what these readings were used for.	Range twice daily to weekly	Weight, BP, HR, O2 saturation, symptoms, ECG, pulse, steps/day, medication, pedal oedema, respiratory effort, facial expressions, ankle circumference	Range 3-12 months
Klersy, 2009	Remote monitoring	NR	NR	Symptoms, weight, BP, physical activity, HR, ECG, arrhythmias, O2 saturation, RV pressure	RCT: Median 6 months (range 2-18 months) Cohort studies: Median 12 months (range 2-17 months)
Maric, 2009	Telemonitoring	Authors report that changes in weight & symptoms were interpreted & appropriate action taken. However, they fail to report what happened in each trial.	NR	Signs, symptoms, weight, medication, BP, CAD risk factors, HR, O2 saturation, QoL, blood test results, pulse, ECG, steps per day	Range 3 months-1 year

First author, year	Type of intervention	Level of patient involvement	Recommended frequency of monitoring	Behaviour/s monitored	Follow-up time
Polinsena, 2010	Telemonitoring	Authors discuss patients being encouraged to assume a more active role in their disease management; however, they fail to report what happened in each trial.	NR	NR	Ranged 30 days-1 year
Inglis, 2010	Telephone support or telemonitoring	It is unclear what happened to the data in a majority of studies. In many, data were sent to a HCP for review; however, it is unclear what happened as a result.	Range twice daily to weekly	Symptoms, adherence, BP, HR, weight, 24hr urine output	Range 3-18 months
Clarke, 2011	Telemonitoring	It is unclear what the data was used for in each trial. 10 studies included physiological monitoring & data were transmitted to HCP to determine if any action was required.	Daily	Weight, heart rate. BP, ECG, HF symptoms	NR
Giamouzis, 2012	Telemonitoring	NR	NR	Weight, BP, heart rate, medication dose, dyspnoea, asthenia or oedema score, blood results, changes in therapy, pulse oximetry, symptoms, 24hr urine output, ECG	Range 6-26 months

**First author, year**	**Type of intervention**	**Level of patient involvement**	**Recommended frequency of monitoring**	**Behaviour/s monitored**	**Follow-up time**
Pandor, 2013	Remote monitoring	NR	Ranged daily to being determined by patient status	Signs & symptoms, current medication, weight, BP, HR, ECG, fluid retention, dyspnoea, ankle circumference, 24hr urine output, o^2 saturation.	Study duration range 3-15 months

BP – Blood Pressure; CAD – Coronary Artery Disease; ECG – electrocardiogram; HCP – healthcare professional; HR – heart rate; HF – heart failure; NR – not reported; RV – right ventricle
